# DUNI: A Portable Smartphone-Coupled Integrating Sphere for Controlled Illumination and Reliable Colorimetric Sensing: Analytical Applications

**DOI:** 10.3390/s26113329

**Published:** 2026-05-24

**Authors:** Pablo Cebrián, José Manuel Escuín, Jesús Salafranca, Carmen Jarne, Ángel López-Molinero, Susana de Marcos, Javier Galbán, Isabel Sanz-Vicente

**Affiliations:** 1 Nanosensors and Bioanalytical Systems (N&SB), Analytical Chemistry Department, University of Zaragoza, 50009 Zaragoza, Spain; cebrianpab@unizar.es (P.C.); gba@unizar.es (J.M.E.); mjarne@unizar.es (C.J.); anlopez@unizar.es (Á.L.-M.); smarcos@unizar.es (S.d.M.); jgalban@unizar.es (J.G.); 2 Analytical Chemistry Department, Aragón Institute of Engineering Research (I3A), University of Zaragoza, 50018 Zaragoza, Spain; fjsl@unizar.es; 3 Aragón Institute of Nanomaterials (INMA), University of Zaragoza-Consejo Superior de Investigaciones Cienctíficas CSIC, 50018 Zaragoza, Spain

**Keywords:** integrating sphere, smartphone, colorimetry, portable device, 3D printing, color analysis

## Abstract

The use of smartphones as analytical instruments is becoming increasingly widespread due to their ease of use and low cost. However, it has limitations, such as dependence on the smartphone’s sensor, the light source and the environment, which hinders the reproducibility and comparability of results. This paper presents the development of a portable device, called DUNI, which can be attached to any smartphone and is designed to overcome these limitations. The device, manufactured using 3D printing and with an average cost of €35, consists of an integrating sphere, which incorporates a lighting-electronic system, as well as accessories for measuring on different surfaces. It has been optimised by evaluating the influence of the optical geometry, the size and reflective coating of the sphere, the lighting conditions, and the electronic stability on measurement performance. It has been applied to the determination of hydrogen peroxide and biogenic amines in synthetic samples, achieving relative errors of less than 5% and detection limits between 3 and 6 µM. Overall, the device we have developed constitutes a portable, versatile and low-cost platform that enables quantitative colorimetric measurements using smartphones under controlled lighting conditions, with potential applications in on-site analysis and resource-limited settings.

## 1. Introduction

Over the past few decades, there has been an increase in the development of alternative, portable, low-cost, user-friendly devices capable of transferring analytical measurements, especially those based on colourimetry, from laboratories [1,2] to field applications and resource-limited settings [3,4]. Within this context, smartphones have emerged as a promising alternative to traditional methods [5,6,7,8,9], since they integrate high-resolution digital cameras, powerful processors, and wireless connectivity. This gives them the potential to be used as accessible optical sensing platforms. Through the analysis of captured images and the extraction of colour parameters (RGB, HSV, among others), smartphones can be transformed into portable photometric devices with transmission capabilities that meet the aforementioned requirements.

However, image-based colourimetric determination presents several limitations that hinder the comparability and reproducibility of results. First, the materials and spectral responses of the colour filters and the CMOS sensors produce significant differences in the recorded RGB values, limiting the comparability between measurements [10,11]. In a previous study, this issue was addressed through the development of software written in Python^®^ v 3.9 that adjusts the colour measurements to standardised conditions (D65 illuminant and sRGB colour space), minimising the differences between RGB values measured with different smartphones. This is achieved by comparing the smartphone’s measurements with the referenced values from an RAL Classic^®^ colour chart [12], which allows the software to generate a correction matrix based on a least-squares fit of the two data sets.

Second, colour acquisition in digital images depends on multiple experimental variables, including the intensity and spectral distribution of the illumination source, the observation angle, the illumination gradient across the sample, and the influence of ambient light [13]. These factors represent some of the most critical limitations for achieving reproducible colorimetric measurements, as they introduce systematic errors and hinder the comparability of results obtained across different laboratories [14].

To minimise these sources of variability, several accessories and complementary devices for smartphones have been developed [15,16,17,18,19]. Most of these devices are based on the concept of a light box [20,21,22], typically consisting of one or more light sources distributed within an enclosure [23]. However, in most of these setups, the illumination is not completely uniform or omnidirectional, resulting in light intensity gradients that may affect measurement accuracy.

A more robust alternative for obtaining homogeneous illumination conditions is the use of integrating spheres. This enables uniform light distribution through multiple diffuse reflections, ensuring that incident light is homogeneously distributed over the internal surface [24,25,26,27]. However, the integration of such systems with smartphones as image acquisition devices remains limited, being mainly focused on optical characterisation or camera calibration applications [25]. Systems combining an integrating sphere with an RGB camera for direct colourimetric image acquisition remain scarce and are mainly restricted to academic prototypes [28]. In these cases, the smartphone is used only as a user interface or data processing platform [29]. No commercial devices have been identified that employ an integrating sphere with the camera of a smartphone for quantitative colour acquisition.

The aim of this work is to develop and optimise a portable integrating-sphere-based device (so-called DUNI) for quantitative colorimetric measurements using a smartphone under homogeneous and controlled illumination conditions. Using an integrating sphere improves the accuracy of the measurements and amplifies the signal obtained compared to using a device without sphere geometry. A compact system manufactured by 3D printing (based on Masked Stereolithography technology, MSLA) was designed, implementing the integrating sphere, the illumination system, the control electronics, and different reaction holders. The influence of the main optical and electronic parameters on measurement precision and reproducibility was experimentally evaluated.

**Figure 1:**
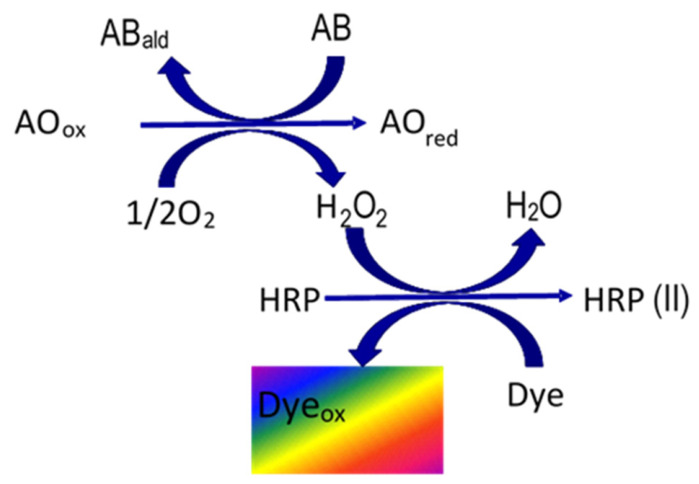
Sequence of enzymatic reactions.

As proof of concept, the device has been applied to the determination of H_2_O_2_ and biogenic amines (AB) (cadaverine, putrescine and tyramine) using a colorimetric enzymatic reaction based on HRP/chromogen (see Figure 1) [30,31] developed on solid supports (test strips and cellulose-based supports). In the case of biogenic amines, a preliminary enzymatic reaction based on the amino oxidase enzyme (AO) is required. To this end, different accessories have been developed that can be attached to the device in order to take measurements on these supports.

## 2. Materials and Methods

### 2.1. Reagents and Solutions

Phosphate buffer solutions (0.1 M, pH 6.0, 7.0 and 8.0) were prepared from Na_2_HPO_4_ and NaH_2_PO_4_ solids (Sigma-Aldrich, Madrid, Spain S9638 and S9763). Hydrogen peroxide stock solution (33% *w*/*v*) was supplied by Panreac, Barcelona, Spain (131077.1211). Cadaverine (C8561), Putrescine (P7505), Tyramine (T287998), 10-Acetyl-3,7-dihydroxyphenoxazine (AR) (90101) and 3,3,5,5-Tetramethylbenzidine (TMB) (860336) were supplied by Sigma-Aldrich, Madrid, Spain. Peroxidase from horseradish (HRP EC 1.11.1.7) was obtained from Sigma-Aldrich, Madrid, Spain (P8125 88.6 Umg-1). Putrescine oxidase (PUO EC 1.4.3.10), 100 μM, was obtained from Gecco Ansterdan, Netherland. Tyramine oxidase (TAO EC 1.4.3.9) from *Arthrobacter* sp. (T-25), 4600 Umg^−1^, was purchased from SEKISUI, Maidston, UK. All solutions were daily weighed and dissolved in the buffer solution ( except TMB and AR), which was dissolved in dimethyl sulfoxide (Panreac131954.1611). TMB and AR solutions were stored in darkness.

Titanium oxide (Daily ART 19105106) and barium sulphate were used for the inner coating of the integrating sphere. Barium sulphate was prepared from 89.2 g dihydrate anhydrous barium chloride (Sigma 217565) and 428 mL of 1M sulphuric acid (7664939 Fisher Scientific, Madrid, Spain). The precipitate was separated by centrifugation, washed repeatedly with distilled water until reaching a pH of 7, and finally dried in an oven, yielding 100 g of BaSO_4_. A 40% (*w*/*w*) solution in water was then prepared. Then, 0.32 g of polyvinyl acetate was added as a binder.

Quantofix™ Peroxid 25 test sticks (from Sigma-Aldrich, Madrid, Spain) and cellulose microcrystalline, 20 μm and average degree of polymerisation less than 350, (Sigma-Aldrich, Madrid, Spain, 310697) were used to prepare sensors for biogenic amines.

### 2.2. Construction of DUNI and Accessories

**Figure 2:**
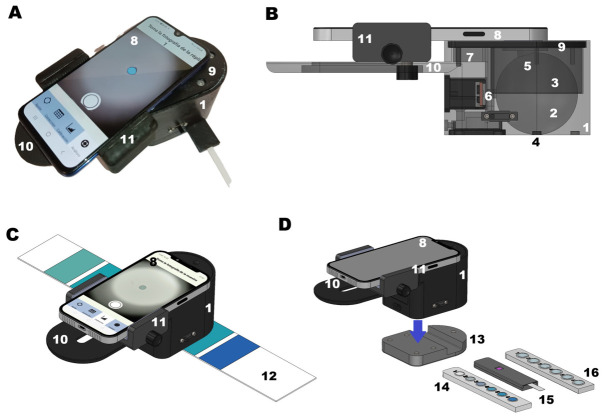
(**A**) Photograph of DUNI device; (**B**) diagram of the components of DUNI (side view). (1) Chassis, (2) lower hemisphere, (3) upper hemisphere, (4) sample port, (5) detector port, (6) battery, (7) LED, (8) charging module, (9) smartphone, (10) support to smartphone, (11) smartphone adapter. (**C**) DUNI with smartphone on an RAL chart (12). (**D**) Accessory (13) for inserting auxiliary components for measurements in solution (14), with strip test (15) or cellulose supports (16).

The device and its accessories (Figure 2) were designed using SolidWorks^®^ software 2023 v31 Dassault Systèmes, París, France) and manufactured using an Elegoo Saturn^®^ resin 3D printer (Elegoo, Shenzhen, China) based on MSLA technology. The prototypes were printed in Elegoo Water Washable Resin Black photopolymer resin. After printing, the parts underwent a washing and curing process using the Elegoo Mercury Plus washing and curing station. The accessories 14, 15 and 16 from Figure 2D were built using Polyethylene Terephthalate Glycol filament (1.75 mm White PETG, SUNLU—SUN-6933582309770) and fabricated by fused deposition modelling (FDM, Bambu Lab P1S). A more detailed description of the device’s components can be found in Supplementary Material MS2.

An airbrush (E4182 Wega Elite, Valencia, Spain ) was used to coat the interior of the integrating sphere with titanium oxide or barium sulphate. The thickness of the layers was measured using a micrometre (Mitutuyo, Amazon, Madrid, Spain).

### 2.3. Digital Imaging Devices

Two devices were used to measure colour: Xiaomi Redmi 6A (13 MPx Camera, Sensor CMOS-Sony IMX486 Exmor RS, f/2.2 aperture) (Xiaomi Inc., Beijing, China) and Xiaomi POCO M4 Pro 5G (50 MPx Camera, Sensor Samsung S5KJN1 ISOCELL, f/1.8 aperture) (Xiaomi Inc., Beijing, China). For all smartphone studies, the camera’s automatic mode was used to take images (the smartphone automatically adjusts settings such as exposure time, ISO sensitivity, white balance and focus), except in Section 3.2.4—Integrating Sphere Coating Materials, where these photographic parameters will have to be modified to evaluate the amount of light captured by the sensor.

### 2.4. Illuminants

Low-power, easy-to-integrate circuit components were used in the design of the lighting control system for the integrating sphere. The charging module used for the device was a TP4056 type C, responsible for charging the internal battery via a type C charger. The lighting system was integrated into a printed circuit board (PCB) incorporating either a W54L511P LED (Roithner Lasertechnik, Vienna, Austria), a white LED (YJHSMT), or an SMD283550S LED (Bridgelux), together with a current stabiliser. The system was powered by a 1000 mAh Lipo battery. The LED power control was implemented via a switch located on one side of the device. The different parts of the system were assembled using 22AWG copper wire (Amazon, Madrid, Spain) connections and soldered with Sn60Pb40 tin.

### 2.5. Digital Imaging Software

The RGB values of the photographic files (JPEG) were analysed with Image J (LOCI, University of Wisconsin, Madison, WI, USA). The RGB values were processed and treated with a self-developed Python software v3.9 in the online platform Project Jupyter. Factorial analysis and data treatment were performed with Microsoft Excel 2010™.

### 2.6. RAL Reference Colorimetric System

Two RAL colour charts (defined by the Deutsches Institut für Gütesicherung und Kennzeichnung, Bonn, Germany; RAL is the acronym for Reichsausschuß für Lieferbedingungen und Gütesicherung) were used to evaluate the various DUNI devices: (a) RAL Classic^®^ colour chart: Collection of 213 colours, each identified by a 4-digit number in combination with the letters “RAL”. This colour chart is referenced in the sRGB colour space and under D65 illuminant conditions. (b) RAL Design^®^ colour chart: Collection of 1688 colours, each identified by a 7-digit number in combination with the acronym “RAL”, based on CIELab model parameters. This colour chart is referenced in the sRGB colour space and under D65 illuminant conditions. To analyse the contrast between the chart and the RGB values obtained with the devices, 96 RAL colour samples were initially characterised and measured using a Konica Minolta CM-2600d spectrophotometer, Valencia, Spain, an instrument that enables the precise acquisition of the hemispheric reflectance spectrum of a surface in the visible range, under d/8 measurement geometry and with control over the exclusion or inclusion of the specular component. This spectrophotometer uses pulsed xenon lamps to illuminate the samples and records their reflectance spectra. This information is then mathematically transformed into the desired colour space and can be compared with the smartphone measurements.

### 2.7. Procedure

#### 2.7.1. Measurement of Coating Spectrum and Whiteness Index

To evaluate the spectral response and reflectance efficiency of the materials, several layers of these were applied to a smooth surface and measured using a Konica Minolta CM-2600d spectrophotometer, Valencia, Spain. The reflective properties of the materials were analysed based on the measured spectra. In the case of white materials, the total amount of reflected light was evaluated using the CIE Whiteness Index, a parameter widely used to quantify the degree of perceived whiteness. This index is calculated from the CIE XYZ tristimulus values derived from the reflectance spectrum, under a standard illuminant (usually D65) and a standard observer. In its simplest form, the whiteness index depends mainly on the luminance value (Y), which represents the total fraction of light reflected by the sample, whilst also incorporating a correction for chromatic deviations from an ideal white (Supplementary Material MS6).

#### 2.7.2. Calculation of the Correction Matrix

To normalise smartphone colour sensor responses relative to a reference system, a colour correction matrix was calculated based on a set of standardised samples. To this end, the nominal RGB values of 55 colour samples from a calibrated RAL Classic chart were used as a reference; these were compared with the RGB values measured by the smartphone using DUNI device. Based on this correspondence between reference and measured values, an adjustment was made using the least-squares method [12], with the aim of estimating a linear transformation matrix that minimises the squared error between the two data sets. This correction matrix is subsequently applied to the sensor measurements, allowing systematic deviations in the camera’s colour response to be compensated for and bringing its outputs closer to the behaviour of the reference system.

#### 2.7.3. Measurement Procedure Using DUNI

Place the smartphone on the DUNI so that the phone’s camera is aligned with the measurement aperture (Figure 2A). Next, switch on the LED and position the DUNI over the sample to be measured (RAL chart, colour strip or cellulose supports). From here, there are two options:

1. Take a photograph, export it to ImageJ v 2.3.0/1.53t to obtain the RGB values, and then to Excel to apply the correction matrix and obtain the normalised RGB values. This option was used throughout the optimisation process.
2. Use a mobile app (developed by INFINITIA Industrial Consulting (https://www.infinitiaresearch.com/ accessed on 15 April 2026)) that integrates all stages of the methodology: image acquisition, extraction of the sample’s RGB values, generation of correction matrices, and normalisation of the measurements using the correction matrix. In addition, colour measurements can be calibrated and interpolated for the quantification of the analytes under study.

To carry out measurements on test strips or cellulose substrates, the accessory shown in Figure 2D must be attached to the DUNI in order to insert the necessary accessories.

All measurements are carried out at least three times, with the result given as the mean and standard deviation.

## 3. Results and Discussion

### 3.1. Device Description

The developed system consists of a compact, fully autonomous integrating-sphere-based device designed for reproducible smartphone colorimetric measurements under controlled illumination. The structure integrates the sphere, illumination system, power supply, and electronic control within a single housing, enabling standalone operation without external accessories. A photograph of the device with the smartphone is shown in Figure 2A. The smartphone screen displays an image of the photograph taken of a sample, from which the RGB coordinates will be obtained.

Figure 2B shows the different parts of the structure designed to house the electronic components, as well as the integrating sphere.

The integrating sphere is formed by two hemispherical parts housed (2) and (3) within a 3D-printed chassis (1). A lower port (4) allows direct placement of the device over the sample, while an upper detection port (5) aligns with the smartphone camera. This configuration ensures that the camera records diffusely reflected light from the sphere interior while minimising the influence of ambient illumination.

Illumination is provided by an internal LED (6) positioned to avoid direct irradiation of the detector or sample, promoting homogeneous diffuse lighting conditions. The device incorporates a rechargeable battery (7) and associated electronics to ensure stable operation of the light source.

The smartphone (8) is placed on top of DUNI (9), aligning the smartphone’s camera with hole 5. To accommodate any smartphone, the device features a protrusion (10) onto which a smartphone adapter (11) can be attached, allowing the smartphone to be secured in place and its camera aligned with the hole (5). The adapter is attached to part (10) using an M5 screw.

Additional construction details, component dimensions, and assembly information are provided in the Supplementary Materials (MS1).

The design is compatible with different smartphone models, as measurements are performed simply by positioning the smartphone camera over the detection port.

The measurement procedure is described in Section 2.7.3. In brief, it involves placing the smartphone on the DUNI so that the phone’s camera is aligned with the measurement aperture. Next, the LED is turned on, the DUNI is placed over the sample to be measured, and the measurement is taken. Figure 2C shows the DUNI with the smartphone attached. It is placed on a standard RAL colour chart, and the image captured by the smartphone of the standard colour can be seen.

In terms of applications, colour can be measured simply by placing the device on the object to be measured (Figure 2C shows DUNI on an RAL chart labelled as 12) or it can be used for analytical determinations based on colorimetric reactions. To this end, an accessory element has been designed (labelled with the number 13 in Figure 2D) that attaches to DUNI, allowing a series of auxiliaries to be inserted that are designed to monitor the reaction in solution (14), on test strips (15) or on cellulose supports manufactured in the laboratory (16).

The selection of the printing material was found to be a critical factor in the performance of the reaction supports. Initial prototypes were fabricated using photopolymer resins; however, these materials produced unexpected interactions with the colorimetric reactions. To eliminate this source of interference, the supports were redesigned using Polyethylene Terephthalate Glycol filament (PETG) and fabricated by fused deposition modelling. PETG provides improved chemical stability, hydrophobicity, and resistance to UV exposure compared with photopolymer resins (Figure S2B in MS1).

### 3.2. Optical Device Design: Design and Optimisation

#### 3.2.1. Mathematical Equation Describing the Device

The light intensity reaching the detector after multiple reflections (I*_R,λ_*) in an integrating sphere is given by

(1) $$I_{R,\lambda }=\frac{I_{0,\lambda }\;f_{d}}{1-\rho \left(1-f_{s}-f_{i}-f_{d}\right)\;}=I_{0,\lambda }f_{d}M$$

(2) $$f_{d}=\frac{A_{d}\;}{A_{T}\;\;\;}\;\;\;\;f_{i}=\frac{A_{i}\;}{A_{T}\;\;\;}\;\;\;\;f_{s}=\frac{A_{s}\;}{A_{T}\;\;\;}$$

where I*_0_,_λ_* represents the incident light and *ρ* is the internal reflectance of the sphere coating. Moreover, *f_d_, f_i_* and *f_s_* are given by Equation (2) and represent the fraction of the total area of the sphere occupied by the detector, illumination system and sample respectively (where *A_d_, A_i_* and *A_s_* represent the detection, illumination and sample areas, respectively, and A_T_ represents the total internal area). Finally, *M* is the sphere multiplier factor and quantifies the amplification of the light intensity inside the integrating sphere due to multiple reflections (compared to the system without sphere). When the integrating sphere is used to measure the reflectance of the light coming from a reflecting sample (*R_λ_* being the reflectance), Equation (1) is modified to

(3) $$I_{R,\lambda }=\frac{I_{0,\lambda }\;f_{d}}{1-\rho \left(1-f_{s}-f_{i}-f_{d}\right)-R_{\lambda }f_{s}\;}=\frac{f_{d}I_{0,\lambda }\;}{f_{s}}\left[\frac{1}{\varphi -R_{\lambda }\;\;}\right]$$

(4) $$\varphi =\frac{1}{Mf_{S}}$$

In the system used in this paper (see later), the sample placed in the *A_S_* of the integrating sphere is illuminated with a polychromatic LED and the reflected light (*I_R_,_λ_*) is collected by a smartphone connected to the sphere, which transforms *I_R_,_λ_* into RGB coordinates. The values of the displayed coordinates (E_(R,G,B)_) are then given by [32] the following equation:

(5) $$E_{\left(R,G,B\right)}=A\frac{f_{d}}{f_{s}}\sum_{\lambda }P_{\lambda }\left[\frac{I_{0,\lambda }\;}{\varphi -\;R_{\lambda }\;\;}\right]$$

where the summation accounts for the discrete spectral response of the CMOS. In this equation, *A* is a constant parameter (including factors depending on the specific camera design, light-to-voltage transformation, image processing and analogue-to-digital conversion) and *P_λ_* is the spectral sensitivity of the CMOS of the smartphone. Considering the broadband nature of the detector spectral response and the absence of sharp features in solids to be measured, the *R_λ_* can be approximated by an effective channel-average reflectance (R_(R,G,B)_), so Equation (5) is simplified to

(6) $$E_{\left(R,G,B\right)}=A\frac{f_{d}}{f_{s}}\frac{1}{\left(\varphi -R_{\left(R,G,B\right)}\right)}\sum_{\lambda }P_{\lambda }I_{0,\lambda }$$

In this section of the paper, we will present the optimisation of the overall optical system involving the smartphone and the integrating sphere device. For clarity, all the parameters compiled in Equation (6) can be grouped into two contributions: sphere (*K_sphere_*) and optoelectronic (*K_oe_*), according to

(7) $$E_{\left(R,G,B\right)}=K_{oe}K_{sphere}\;K_{sphere}=\frac{f_{d}}{f_{s}}\frac{1}{\left(\varphi -R_{\left(R,G,B\right)}\right)}\;\;\;\;\;\;\;{\;K}_{oe}=A\sum_{\lambda }P_{\lambda }I_{0,\lambda }\;$$

These parameters guided the design of the DUNI device.

#### 3.2.2. Experimental Procedure for Evaluating the Device

The aim of this study is to fabricate an integrating sphere which, whilst maintaining dimensions appropriate to the size of the device to be manufactured, allows for the greatest possible signal amplification. Furthermore, when light enters through an aperture in the sphere, it is not initially distributed uniformly within it, but retains its direction of entry. Under these conditions, the amount of light incident on the sample depends largely on the geometry of the sphere. For the integrating sphere to function as such, the light must undergo multiple reflections within it. This process homogenises the radiation, causing it to lose the memory of its initial direction. Only when this regime is reached do the fractions of light striking the sample follow Equation (3). In other words, the aim is also for the radiation within the sphere to achieve the greatest possible degree of homogenisation.

To this end, standardised colour references were used to evaluate and optimise the device’s RGB response. Specifically, commercially available RAL colour strips with certified RGB values were used as calibration standards, enabling a direct comparison between the measured coordinates and the reference values. We assessed the agreement between measured and certified RGB values by linear regression analysis. The slope provides a direct measure of the amplification achieved by the system, with higher values indicating greater optical throughput. The intercept offers complementary information, particularly when it increases proportionally with the slope, reflecting a consistent offset in the amplification process. The coefficient of determination (R^2^) was used as an operational measure of the homogeneity of the internal illumination, as higher values reflect a more linear and geometry-independent response.

#### 3.2.3. K_sphere_ Optimisation

1. Smartphone Positioning and Stabilisation System

Variations in smartphone positioning during image acquisition can introduce significant variability in colorimetric measurements due to small changes in camera–sample distance, viewing angle, and alignment with the detection port. To minimise this source of experimental uncertainty, a mechanical stabilisation system was integrated into the device (Figure 2B, labelled as 10 and 11). The system consists of a dedicated mounting extension incorporated into the device structure and a smartphone holder that allows precise alignment of the camera with the detection port. Once positioned, the holder can be fixed using a pressure screw, ensuring stable and reproducible image acquisition.

To evaluate this effect, five replicate images of an RAL colour reference sample were acquired under identical illumination conditions, both with and without mechanical stabilisation of the smartphone (Supplementary Material MS2). Figure S3 shows the superposition of the acquired images in both cases, illustrating the increased positional variability observed when no stabilisation system is used. The corresponding RGB values are summarised in Table S1. Relatively large dispersion was observed in all colour channels, with standard deviation values of approximately 7–8 RGB units.

This design feature contributes to reducing operator-dependent variability and enhances the reliability of the device for portable analytical applications. It also allows connection to any smartphone.

2. Port Geometry: Detector Angle (0° vs. 8°)

The relative angle between the detection port (smartphone camera) and the sample surface inside an integrating sphere can significantly influence the accuracy and quality of optical colour measurements, particularly due to the contribution of specular reflection components (Supplementary Material MS3, ref. [33]). To evaluate this effect, measurements were performed with the detector positioned at 0° and 8° relative to the sample’s normal, corresponding to internal diffuse illumination geometries d/0 and d/8, respectively.

**Figure 3:**
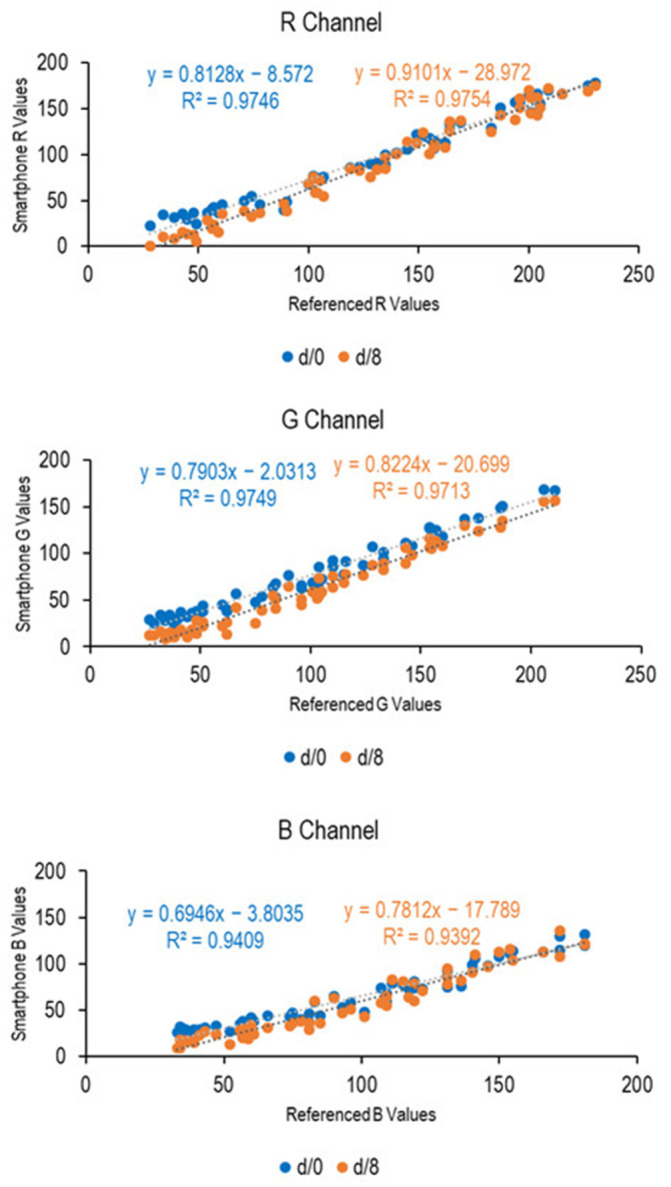
Correlation between the RGB values measured with the Smartphone-DUNI and the actual values of the patterns. Each graph shows the results obtained for each channel comparing between the device with d/0 and d/8 geometry. Photographic conditions of measurements: Automatic mode enabled and focal length 10 mm.

Figure 3 shows the correlation between the measured RGB values (*y*-axis) and the nominal values (*x*-axis) of 55 RAL colour standards. Across all channels, the d/8 configuration produced slopes closer to unity compared with the d/0 geometry, indicating a response that is more proportional to the reference RGB values. The intercepts obtained for d/8 were more negative, which can be attributed to the exclusion of specular reflection components in this geometry. While the d/0 configuration collects both diffuse and specular reflected light, the d/8 arrangement primarily measures diffuse reflectance, resulting in more consistent colorimetric measurements.

In both configurations, greater dispersion was observed for lower RGB values, corresponding to highly saturated colours.

Additionally, the influence of detector angle on spatial colour homogeneity was evaluated through image analysis, using ImageJ v 2.3.0/1.53t (Supplementary Material MS4, ref. [34]). Intensity profiles extracted from the measurement region (Figure S5) showed improved spatial uniformity for the d/8 configuration (the standard deviation of the values was 0.65 with an angle of 8°, while with 0° it was 5.64). This suggests more homogeneous diffuse illumination inside the integrating sphere, which contributes to enhanced repeatability and reliability of the colorimetric measurements.

Based on these results, the d/8 geometry was selected for subsequent experiments.

3. Integrating Sphere Size

The dimensions of the sphere, including total area (*A_T_*, i.e., the size of the sphere), and the areas of the three ports (*A_s_, A_d_, A_i_*) are very important parameters affecting its performance. To optimise these parameters, the port areas were fixed according to the sizes of the test strip (*A_s_*), detector (*A_d_*) and LED (*A_i_*), and the total area *A_T_* was optimised. The sphere model predicts that increasing the total area, while keeping the dimensions of the individual ports constant, would result in a reduction in the amount of light reaching the detector. However, as mentioned above, the geometry of the sphere must be appropriate to obtain the associated benefits; therefore, smaller spheres may not behave as expected, resulting in lower amplification and non-uniform light distribution. It is only when this regime is reached that the fractions of light striking the sample follow Equation (2). This behaviour does not occur abruptly, but progressively.

According to this, four integrating sphere diameters (33, 36, 39, and 42 mm) were evaluated. The dimensions and positions of all ports were kept constant for all devices, with diameters of 6 mm for the detection port, 4 mm for the illumination port, and 8 mm for the sample port.

Experimental performance was evaluated using 24 RAL colour standards (Tables S2–S4 in Supplementary Material MS5). Correlations between measured RGB values and nominal reference values showed that, prior to correction, larger sphere diameters generally produced higher slopes, intercepts, and correlation coefficients, indicating improved light collection efficiency.

Considering theoretical efficiency, experimental consistency, and practical integration of electronic components, the 42 mm integrating sphere was selected as the optimal configuration for the final DUNI device.

4. Integrating Sphere Coating Material

According to Equation (6), the higher the ρ, the higher the E_(R,G,B)_. To evaluate this common strategy to enhance diffuse reflectance on optical surfaces, consisting of applying a granular white coating that promotes multiple light scattering events, two coating materials were evaluated for the inner surface of the integrating sphere: barium sulphate (BaSO_4_) and titanium dioxide (TiO_2_) (Supplementary Material MS6, ref. [35]).

In this case the reflectance spectra were obtained. The results revealed distinct optical behaviours for both materials. Titanium dioxide exhibited a noticeable absorption band in the near-UV region (approximately 360–400 nm; Figure S6A), whereas barium sulphate showed a more spectrally homogeneous response across the visible range (Figure S6B). Quantitative comparison of total reflected light indicated reflectance values of approximately 89% for TiO_2_ and 97% for BaSO_4_. These results indicate that BaSO_4_ provides both higher overall reflectance and better spectral uniformity, two critical factors for minimising systematic bias in colorimetric measurements. Consequently, BaSO_4_ was selected as the most suitable coating material for the integrating sphere.

The influence of coating thickness was then investigated by applying successive BaSO_4_ layers to the surface (Figure S7). Reflectance and whiteness index increased progressively with the number of layers, reaching a plateau around the seventh layer. From a practical standpoint, additional layers beyond this threshold provide negligible optical benefit while increasing preparation time and material consumption.

Reproducibility of the coating procedure was also evaluated. Three independent DUNI devices were coated with seven BaSO_4_ layers using an airbrush application protocol (Table S5). One-way ANOVA analysis revealed no statistically significant differences among the devices, demonstrating that the coating method is reliable and yields consistent optical properties across independently prepared units.

Finally, the effect of a protective varnish layer on optical performance was assessed (Figure S8, ref. [36]). The main effect was limited to the near-UV region, with minimal influence across the visible spectrum. The varnish layer nonetheless provides mechanical protection and improved resistance to UV exposure, which are important for long-term device stability.

Overall, these results indicate that BaSO_4_ is the most appropriate coating material for the integrating sphere. Optimal performance is achieved with seven airbrushed layers, optionally protected with a varnish coating to enhance durability without significantly compromising optical performance.

#### 3.2.4. *K_oe_* Optimisation

1. Illuminant

The device is designed to be used with any mobile device. According to Equations (6) and (7), the E_(R,G,B)_ obtained for a given sample will depend on the spectral response of the CMOS sensor and the type of light source. As the light source is built into the device, the one that provides the best results can be selected. To do that, three LED illuminants with different spectral emission characteristics were selected as representative of the most commonly used illuminants: W54L511P (≈6500 K), YJHSMY (≈5000 K) and SMD283550S (≈5000 K). Their main differences lie in their spectral power distributions (Supplementary Material MS7, Figure S9A–C), particularly in the relative intensity of the blue region of the spectrum.

**Figure 4:**
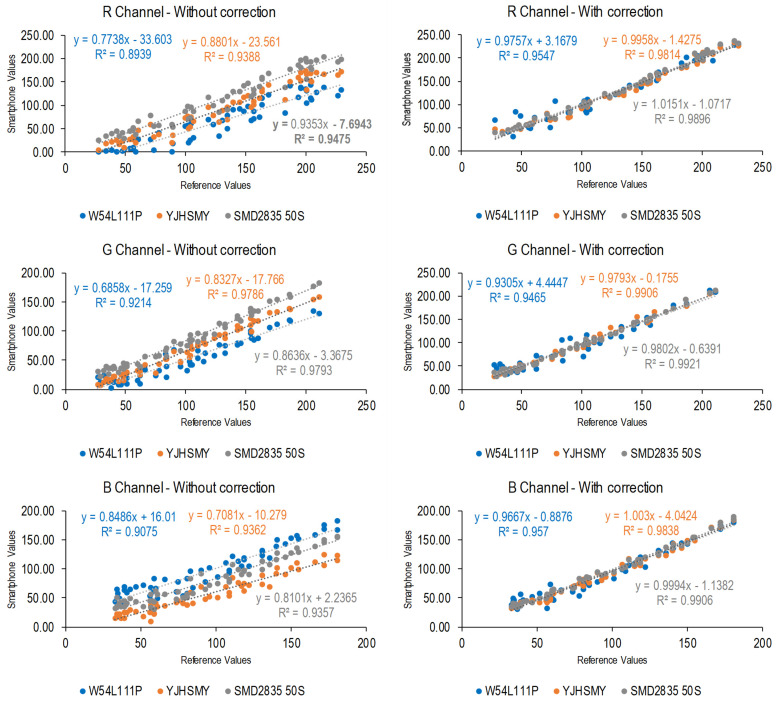
Correlation between the RGB values measured with the smartphone and DUNI and the actual values of the patterns. Each graph shows the results obtained for each channel with each illuminant. (**Left**) Before correction, (**Right**) after correction. Photographic conditions of measurements: Automatic mode enabled and focal length 10 mm.

Colour measurements were obtained from 55 RAL reference colour samples. The images acquired with those LEDs are shown in Figures S10–S12. Figure 4 shows the correlations between the RGB values measured using the smartphone camera and the corresponding standardised RAL reference values.

In short, all three components perform best with the illuminant SMD2835 50S. However, the intercepts in the R and G channels are negative for the three illuminants, whereas in the B channel, the behaviour differs: in the case of the YJHSMY illuminant it is negative and in the case of W54L111P and SMD2835 50S it is positive. This behaviour can be justified not only by the spectral distribution of the different illuminants used, but also by the internal signal processing of the smartphone camera, including offset corrections and channel-dependent adjustments.

The W54L111P illuminant, with higher spectral content in the blue region, causes compensation by the smartphone’s internal algorithm in the blue channel, raising the average B values, which generates a more positive origin than the other illuminants. On the other hand, the YJHSMY LED, with less predominance in the blue region compared to other areas of the spectrum, causes the device’s internal algorithms to make less significant compensations between what the sensor captures and how the image is displayed. This same effect is observed in the SMD2835 50 S LED; however, due to its more continuous spectral emission, the compensations in all channels are much lower than in the other two illuminants. These can be seen in a slope closer to 1 and the intercepts closer to 0.

These results confirm that the illuminant significantly influences raw smartphone-based colour measurements. Internal camera processing tends to attenuate spectral differences between light sources, but residual effects remain and can impact quantitative colour analysis. Consequently, careful selection and characterisation of the illumination source are essential for improving accuracy and reproducibility in smartphone colorimetric applications.

2. Smartphone CMOS: Correction matrix

Each smartphone incorporates a specific CMOS sensor, characterised by its own spectral response function *P_λ_* (see Equations (6) and (7)). To standardise measurements across different devices, a correction matrix was computed to compensate for sensor-dependent differences and enable comparability of RGB values obtained with different smartphones. This matrix is determined individually for each smartphone following the procedure described in Section 2.7.2. The correction matrix also depends on the illuminant. When the LED is changed and a new matrix is computed, the combined spectral response of the sensor–illuminant system is effectively compensated, allowing RGB values acquired with different smartphones and LEDs to be directly comparable. Figure 4 also shows the correlation lines between the measured RGB and the standardised RAL RGB values for the three different LEDs when the appropriate correction matrix is applied in each case. These results demonstrate the effectiveness of the correction procedure.

After applying the correction matrix, the term $\sum_{\lambda }P_{\lambda }I_{0,\lambda }$ can be treated as an effective constant that is consistent across different smartphones. This allows Equations (6) and (7) to be simplified as follows:

(8) $$E_{\left(R,G,B\right)}=B\frac{f_{d}}{f_{s}}\frac{1}{\left(\varphi -\;R_{\left(R,G,B\right)}\right)\;}=K_{oe}K_{sphere}K_{oe}=B$$

3. Electronics and Autonomy

LED luminous intensity depends directly on the electrical current; therefore, fluctuations in the power supply can affect illumination stability and, consequently, colorimetric measurements. To evaluate this effect, the illumination inside the integrating sphere of the DUNI device was monitored during battery discharge, both without current stabilisation (WEC) and with an integrated current stabiliser circuit (CEC) (Supplementary Material MS8).

The results show that, without current stabilisation (Figure S13A), battery discharge leads to a gradual decrease in the current and a corresponding reduction in luminous intensity, approximately following an exponential decay. This behaviour introduces a source of systematic error in colorimetric measurements, as illumination conditions change over time, unless a new correction matrix is recalculated and applied.

In contrast, with stabilisation (Figure S13B), the current remains nearly constant throughout most of the battery discharge cycle, resulting in stable illumination until a sharp drop occurs near depletion. This behaviour ensures more controlled and reproducible measurement conditions.

The impact on colour measurements was evaluated using five blue RAL samples with different tonalities measured repeatedly throughout the battery discharge cycle (Supplementary Material MS8, Figure S14). Without stabilisation, RGB values show systematic drift with battery level, whereas with stabilisation they remain significantly more stable.

These results confirm that current stabilisation is essential for reliable and reproducible measurements.

Based on these optimisation studies, the final configuration of the DUNI device consists of a 42 mm diameter integrating sphere with d/8 measurement geometry, illuminated by an LED SMD2835 50 S powered through a current stabilisation circuit.

All subsequent measurements described in this work were performed using this optimised configuration.

The repeatability and reproducibility of measurements taken with the DUNI device were evaluated. To this end, the RGB coordinates of an RAL standard (210-70-20) were measured (*n* = 5) over a period of 10 days. The results, expressed as %RSD for each RGB coordinate, were a repeatability of 0.9, 0.6 and 0.6, and a reproducibility of 1.2, 0.9 and 0.8.

#### 3.2.5. Cost Assessment

The total mass of the device was approximately 250 g, corresponding to a resin cost of approximately 6 €, based on a commercial photopolymer resin price of 24 € per kg, and an additional printing cost of approximately €4 for a 12 h printing time. Additional costs included standard fastening elements (M2/M3 screws and threaded inserts), estimated at €4, and electronic components such as the LED PCB, charging module and power supply, with a combined cost of approximately €15. The overall manufacturing cost of the complete device prototype was therefore estimated at approximately €25–35, excluding labour costs and equipment depreciation. This low-cost fabrication approach enables rapid prototyping and facilitates the reproducibility of the system in other laboratories.

### 3.3. Application of the Sphere Device to Analytical Quantification

#### 3.3.1. Mathematical Equation Describing the Quantification Performance

The Kubelka–Munk (KM) [37,38] equation is a phenomenological, deterministic, continuous model widely used to relate the transmitted and reflected light (*R_λ_*) of a solid of thickness l to two main parameters: the KM absorption coefficient (*K_KM,λ_*) and the KM scattering coefficient (*S_KM,λ_*). Despite the existence of several models describing the relationship between both *K_KM,λ_* and *S_KM,λ_* and the intrinsic absorption (*K_λ_*) and scattering (*S_λ_*) coefficients, a convenient relationship for an anisotropic medium is

(9) $$K_{KM,\lambda }=K_{\lambda }=2.3\sum_{i}\varepsilon_{\lambda ,i}C_{i}\;\;\;\;\;\;\;\;\;\;\;\;\;\;S_{KM,\lambda }=\frac{S_{\lambda }}{2}$$

where *C_i_* and *ε_λ,i_* are the concentration and the molar absorptivity of each absorbing species in the medium. The most general expression for *R_λ_* derived from the *KM* equation is

(10) $$R_{\lambda }=\frac{Sinh\;(b_{\lambda }S_{KM,\lambda }l)}{a_{\lambda }Sinh\;\left(b_{\lambda }S_{KM,\lambda }l\right)+b_{\lambda }Cosh\;(b_{\lambda }S_{KM,\lambda }l)}$$

where

(11) $$a_{\lambda }=1+\frac{K_{KM,\lambda }}{S_{KM,\lambda }}\;\;\;\;\;\;\;\;\;\;\;b_{\lambda }=\sqrt{a_{\lambda }^{2}-1}$$

By combining Equations (8)–(11) an expression of the form

(12) $$E_{\left(R,G,B\right)}=f(\sum_{i}\varepsilon_{\lambda ,i}C_{i})$$

is obtained. In the devices used in this work, only one absorbing species is present. What is observed experimentally is that *R_λ_* varies with *C* according to a second-degree polynomial function. Therefore, the final expression is approximated by a Taylor expansion series (see Supplementary Material MS9). Assuming (1) constant S over the spectral range, (2) thin-medium approximation (Sl < 1), and (3) small fs value (fs << 1), the final expression becomes

(13) $$E_{\left(R,G,B\right)}=E_{0,(R,G,B)}\left(1-\alpha C+{\beta C}^{2}\;\right)$$

(14) $$E_{0,(R,G,B)}=B\frac{f_{d}}{f_{s}}\frac{1}{\left(\varphi -\frac{Sl}{Sl+2}\right)\;}\;\;\;\;\;\alpha =1.15\frac{f_{s}Sl^{2}}{\left(\varphi -\frac{Sl}{Sl+2}\right)}\varepsilon \;\;\;\;\;\;\beta =1.8\frac{f_{s}Sl^{3}}{\left(\varphi -\frac{Sl}{Sl+2}\right)}\varepsilon^{2}$$

where the E_0,(R,G,B)_ is the value of the corresponding coordinate in the absence of the absorbing species. Although E_(R,G,B)_ can be used directly as the analytical signal, improved accuracy is obtained by normalising with respect to the blank signal E_0,(R,G,B)_.

(15) $$\frac{{E_{0,(R,G,B)}-E}_{\left(R,G,B\right)}}{E_{0,(R,G,B)}}=\alpha C-{\beta C}^{2}$$

This parameter corrects for variations associated with smartphone design while preserving the sensitivity enhancement provided by the integrating sphere geometry.

#### 3.3.2. Smartphone App for Quantification

To facilitate data acquisition and analytical processing, a dedicated smartphone application was developed as an integral component of the DUNI system. The app enables standardised image capture, automatic extraction of RGB values from selected regions of interest, and correction of colour coordinates using the previously described calibration algorithm. In addition, stored calibration models (such as described in Equation (15)) can be applied directly to calculate analyte concentrations in real time. All measurement data are automatically saved within the application and can be exported in standard formats for further statistical analysis or traceability. This digital integration transforms DUNI into a fully portable and self-contained colorimetric analysis platform.

The DUNI device was conceived as a quantitative colorimetric analysis platform compatible with different reaction formats. Since colorimetric assays can be performed on diverse supports—including liquid solutions, paper-based materials, and commercial reactive strips—an accessory was developed to allow interchangeable sample holders while preserving the optical geometry of the device (labelled as 13 in Figure 2D).

This modular configuration expands the applicability of the DUNI device without modifying its optical configuration or illumination conditions. In this section, the analytical performance obtained using some of these accessories is presented.

#### 3.3.3. Commercial Reactive Strips

For measurements based on commercial test strips, the auxiliary holder shown in Figure 2D, labelled as 15 (and described in Supplementary Material MS1B), was inserted into the accessory element presented in Figure 2D, labelled as 13, ensuring fixed positioning and reproducible optical geometry.

1. Determination of Hydrogen Peroxide

Numerous peroxide test strips are commercially available; in this work, Quantofix Peroxide 25^®^ test strips were used. These strips contain a horseradish peroxidase and 3,3′,5,5′-tetramethylbenzidine (TMB) as dye for the redox reaction. The reaction is shown in the scheme in Figure 1.

The colour transition of the strips occurs within the range 0–25 mg/L H_2_O_2_, producing a cyan-like colour scale.

**Figure 5:**
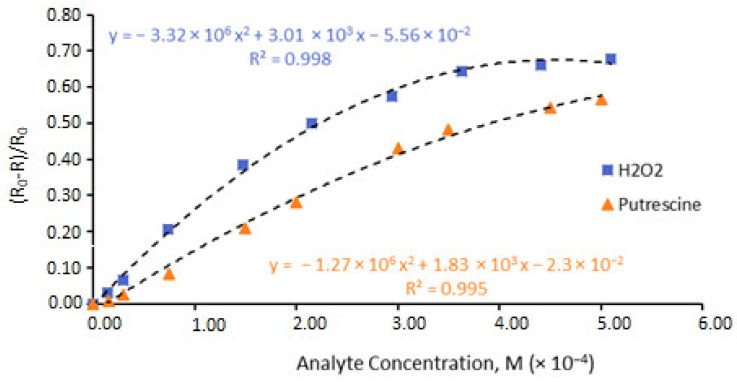
Calibration curve using DUNI and commercial reactive strips for hydrogen peroxide (squares) and putrescine (triangles). Conditions: 0.1 M phosphate buffer, pH = 7.

Ten standard H_2_O_2_ solutions of known concentration were prepared. Each solution was applied to the strips, and measurements were performed after 15 s using the smartphone coupled to DUNI. The R coordinate was selected as an analytical signal due to the absorption characteristics of the oxidised chromophore around 650 nm. The calibration plot of (R_0_ − R)/R_0_ versus H_2_O_2_ concentration is shown in Figure 5 (squares). As previously demonstrated, a second-order polynomial relationship between (R_0_ − R)/R_0_ and chromophore concentration is theoretically expected.

The analytical characteristics are shown in Table 1 (row A).

Four additional peroxide solutions were prepared and interpolated in the calibration curve. Each sample was measured in triplicate. The obtained results are summarised in Table 2 (row A) with errors of less than 2%.

These results can also be used to validate the mathematical model given in Equation (15). When the coefficient of the C2 term (*β*) is divided by the coefficient of the C term (*α*),

(16) $$\;\;\frac{\beta }{\alpha }=1.5l\bar{\varepsilon }=1102$$

which is consistent with the averaged molar absorptivity of oxidised TMB in the R coordinate spectral zone (~35,000 M^−1^cm^−1^) and the average width of the test strip (~0.2 mm). Moreover, considering that the f value can be calculated from the port dimensions and the reflectance of the BaSO_4_ (f = 0.045), the S value for the test strip material can be calculated, giving S = 4.4 cm^−1^, which is consistent with values reported in the literature [39].

2. Determination of Biogenic Amines: Putrescine and Cadaverine

These peroxide-sensitive strips can also be employed for analytes whose enzymatic oxidation produces hydrogen peroxide as a reaction product. Biogenic amines such as putrescine represent a relevant example, since their oxidation by amine oxidases generates H_2_O_2_ (Figure 1).

Putrescine determination was carried out after reaction with putrescine oxidase (PUO). For this purpose, the commercial strips were pre-treated by depositing 10 µL of PUO solution (25 μM).

Ten standard putrescine solutions were prepared and applied to the modified strips. The signal was recorded 120 s after injection. The corresponding calibration curve is shown in Figure 5 (triangles) and the analytical characteristics are shown in Table 1 (row B).

If, in this case, the coefficient of the C^2^ term (β) is divided by the coefficient of the C term (α), the result is 694, which is approximately 70% lower than the theoretical value; this is consistent with the difference in sensitivity observed in solution [31].

Four additional putrescine solutions were prepared and interpolated in the calibration plot. Each solution was measured in triplicate. The results are summarised in Table 2 (row B) with errors of less than 4%.

When applying the same methodology for cadaverine determination, no measurable signal was observed below 3 × 10^−4^ M (Figure S15 in Supplementary Material MS10).

Although commercial strips offer convenience and rapid implementation, their main limitation lies in the unknown composition and concentration of the immobilised reagents. This constraint highlights the importance of developing customisable reactive platforms with controlled composition.

#### 3.3.4. Home-Made Cellulose Supports: Determination of Cadaverine

To overcome the limitations associated with commercial strips, laboratory-prepared cellulose-based supports were developed. The research group has extensive experience in the preparation of paper-based analytical devices, allowing controlled immobilisation of enzymes and chromogenic reagents.

For use within DUNI, the auxiliary mould shown in Figure 2D (labelled as 16) and S2C was employed. This mould allows fabrication of cellulose reaction wells that can be directly inserted into the accessory labelled as 13 in Figure 2D, maintaining fixed geometry during image acquisition.

After optimisation (Supplementary Material MS10), each well was prepared by depositing 75 µL of a 5% cellulose solution containing TMB (6 × 10^−4^ M) and allowing it to dry for 1 h. Subsequently, 10 µL of a mixture containing PUO (25 µM) and HRP (10 U/mL) was added, followed by 10 µL of cadaverine standard solutions. The corresponding calibration curve is shown in Figure S18.

Corrected RGB values were obtained, and the R coordinate was selected as the analytical parameter. The resulting calibration curve demonstrated successful cadaverine determination under controlled conditions with quantitative results summarised in Table 1 row C.

Two additional cadaverine solutions were prepared and interpolated in the calibration plot. Each solution was measured in triplicate. The results are summarised in Table 2 (row C) with errors of less than 3%.

The fabrication of cellulose-based supports enabled reliable cadaverine quantification. Moreover, these platforms can also be employed for putrescine and hydrogen peroxide determination (in the latter case without the need for PUO immobilisation), demonstrating the versatility of the DUNI system.

#### 3.3.5. Other Determinations

Cellulose-based supports can be manufactured according to the desired application, as it is possible to immobilise other enzymes and other dyes (Supplementary Material MS11), highlighting the versatility of the device and methodology.

1. Tyramine was determined by immobilising tyramine oxidase (10 µL of 12 U mL^−1^). The corresponding calibration curve is shown in Figure S19 and the analytical characteristics in Table 1 (row D). Four samples were interpolated, yielding the results shown in Table 2 row D with errors of less than 2%.
2. Other dyes, such as Amplex Red, which is pink in its oxidised form, can be immobilised, highlighting the possibilities of using other colour ranges. Amplex Red was immobilised to determine H_2_O_2_ and cadaverine. In this case, coordinate G was chosen as it showed the greatest variability with respect to the analyte concentration. The calibration graphs are shown Supplementary Material MS11 in Figures S20 (H_2_O_2_) and S21 (cadaverine). The characteristics of the method are found in Table 1, rows E and F, and the results of sample determination in Table 2, rows E and F, to H_2_O_2_ and cadaverine respectively.

### 3.4. Comparison of DUNI with Light-Box

The ΔE2000 and RGB RMSE values were used to compare the DUNI device with the Light-Box employed in previous studies. These values were calculated using the complete set of 55 RAL reference patterns after applying the correction matrices [12]. ΔE2000 is a mathematical formula used in the CIE Lab colour space to quantify the perceptual difference between measured and reference colour values. Lower ΔE2000 values indicate greater colour accuracy and a closer match to the original colour standard, whereas higher values indicate more noticeable colour differences. RMSE values represent the residual RGB error between the corrected measurements and the corresponding sRGB-D65 RAL reference values.

The results (Table 3) demonstrate that the integrating sphere configuration provides improved colorimetric performance and measurement reproducibility under controlled acquisition conditions. Specifically, lower mean ΔE2000 values were obtained (1.89 vs. 2.55), together with lower RGB RMSE values.

The enzymatic determination of H_2_O_2_ using commercial test strips was performed with the DUNI and with Light-Box. The analytical characteristics are shown in Table 1, for rows A and G, respectively. As can be seen, improvements were observed in both sensitivity and the limit of detection (LoD) using DUNI.

## 4. Conclusions

A portable smartphone-based colorimetric device (DUNI) incorporating an integrating sphere was developed and experimentally optimised. The influence of optical geometry, integrating sphere size, illumination conditions and electronic stability on measurement performance was systematically evaluated.

The optimised configuration, consisting of a 42 mm integrating sphere with d/8 geometry, stabilised LED illumination and controlled smartphone positioning, provided accurate and reproducible colour measurements. The application of a matrix-based correction procedure further improved agreement with standardised colour references, achieving linear responses and relative errors below 5%.

Moreover, mathematical models describing the behaviour of the analytical system have been developed. The proposed device represents a simple and low-cost platform for quantitative colorimetric analysis and shows potential to overcome contextual influences, thereby facilitating the comparison of colour images acquired under reference conditions. This capability makes it suitable for implementation in high-throughput analytical applications such as food quality control, environmental monitoring, and point-of-care testing.

## Figures and Tables

**Table 1:** Analytical characteristics. Row A–F: Using DUNI. Row G: Using Light-Box (E represents R or G or B coordinate, LoD = limit of detection, RSD = relative standard deviation).

	Support /Dye	Analyte	Calibration Line (E_0_ − E/E_0_) = K_2_C^2^ + K_1_C + K_0_	Range (M)	LoD (M)	RSD % (*n*)
A	Strip test /TMB	H_2_O_2_	K_2_ = −3.32 × 10^6^; K_1_ = 3.01 × 10^3^; K_0_ = −5.56 × 10^−3^	1.5 × 10^−5^–5.1 × 10^−4^	3.3 × 10^−6^	4% (5)
B	Strip test /TMB	Putrescine	K_2_ = −1.27 × 10^6^; K_1_ = 1.83 × 10^3^; K_0_ = −2.30 × 10^−3^	2.0 × 10^−5^–6.0 × 10^−4^	5.9 × 10^−6^	5% (5)
C	Cellulose /TMB	Cadaverine	K_2_ = −2.27 × 10^6^; K_1_ = 3.42 × 10^3^; K_0_ = −2.49 × 10^−2^	2.5 × 10^−5^–4.0 × 10^−4^	9.9 × 10^−6^	5% (5)
D	Cellulose /TMB	Tyramine	K_2_ = −3.80 × 10^6^; K_1_ = 3.42 × 10^3^; K_0_ = −2.49 × 10^−3^	2.5 × 10^−5^–3.0 × 10^−4^	6.6 × 10^−6^	5% (5)
E	Cellulose /AR	H_2_O_2_	K_2_ = −2.44 × 10^6^; K_1_ = 2.07 × 10^3^; K_0_ = 6.60 × 10^−3^	1.5 × 10^−5^–5.1 × 10^−4^	5.5 × 10^−6^	5% (5)
F	Cellulose /AR	Cadaverine	K_2_ = −5.85 × 10^6^; K_1_ = 3.18 × 10^3^; K_0_ = 1.32 × 10^−2^	1.0 × 10^−5^–3.0 × 10^−4^	5.2 × 10^−6^	5% (5)
G	Strip test /TMB [12]	H_2_O_2_	K_2_ = −1.62 × 10^6^; K_1_ = 1.94 × 10^3^; K_0_ = −1.79 × 10^−2^	1.5 × 10^−5^–5.1 × 10^−4^	5.8 × 10^−6^	5% (5)

**Table 2:** Results of application to synthetic samples.

Application	Support/Dye	Analyte	Real Concentration (mol/L)	Found Concentration ± s (mol/L)	Relative Error (%)
A	Commercial	H_2_O_2_	4.41 × 10^−5^	(4.35 ± 0.03) × 10^−5^	−1.4
	Strip test/TMB		7.50 × 10^−5^	(7.52 ± 0.09) × 10^−5^	0.2
			1.35 × 10^−4^	(1.32 ± 0.41) × 10^−5^	−2.5
B	Commercial	Putrescine	4.00 × 10^−5^	(4.20 ± 0.13) × 10^−5^	5.0
	Strip test/TMB		8.00 × 10^−5^	(7.70 ± 0.31) × 10^−5^	−3.7
			2.25 × 10^−4^	(2.19 ± 0.51) × 10^−5^	−2.8
C	Cellulose/TMB	Cadaverine	8.00 × 10^−5^	(7.72 ± 0.53) × 10^−5^	−3.5
			1.25 × 10^−4^	(1.24 ± 0.03) × 10^−4^	−0.8
D	Cellulose/TMB	Tyramine	1.25 × 10^−4^	(1.21 ± 0.17) × 10^−4^	−3.2
			1.75 × 10^−4^	(1.76 ± 0.51) × 10^−5^	0.2
E	Cellulose/AR	H_2_O_2_	5.00 × 10^−5^	(4.55 ± 0.31) × 10^−5^	−8.9
			8.10 × 10^−5^	(8.17 ± 0.37) × 10^−5^	0.8
			1.69 × 10^−4^	(1.66 ± 0.23) × 10^−4^	−2.0
			2.76 × 10^−4^	(2.19 ± 0.40) × 10^−5^	−4.3
F	Cellulose/AR	Cadaverine	4.00 × 10^−5^	(3.92 ± 0.26) × 10^−5^	−2.0
			6.00 × 10^−5^	(5.91 ± 0.22) × 10^−5^	−1.4
			9.00 × 10^−5^	(9.14 ± 0.15) × 10^−5^	1.6
			1.25 × 10^−4^	(1.20 ± 0.25) × 10^−5^	−3.4

s: Standard deviation (n = 3).

**Table 3:** Comparison between Light-Box and DUNI.

Metric/Parameter	Light-Box System [12]	Integrating Sphere System (Current Work)
**Illumination type**	LED Strip	PCB LED with current stabilisation
**Mean ΔE2000 (1:1:1) after correction (55 RAL patterns)**	2.55	1.89
**Minimum ΔE2000 (1:1:1) after correction**	0.4216	0.1208
**RGB RMSE vs. RAL references (after correction)**	R: 9.95/G: 6.73/B: 6.33	R: 6.47/G: 5.12/B: 5.57

## Data Availability

No data was used for the research described in the article.

## Supplementary Materials

The following supporting information can be downloaded at https://www.mdpi.com/article/10.3390/s26113329/s1: MS1: Device design and auxiliary components; Figure S1: Cross-section of the DUNI device; Figure S2: (A) Component parts and assembly of the holder for commercial test strips, (B) Variation of the R coordinate over time in a blank test using a test strip holder made of the polymer resin and PETG and (C) Preparation of cellulose supports; MS2: Need for a position stabiliser; Figure S3: Superimposition of 5 images taken: (A) without a stabiliser and (B) with a smartphone stabiliser. Table S1: Measurements of five replicas of an RAL standard, with and without a smartphone stabilisation system; MS3: Angle effect; Figure S4: Most common geometries used for integrating spheres; MS4: ImageJ v 2.3.0/1.53t software to study colour homogeneity; Figure S5: Photograph of an RAL standard taken with the d/0 (A) and d/8 (B) device, and light distribution across the surface of the d/0 (C) and d/8 (D); MS5: Integrating sphere size; Table S2: Correlation between each device’s measurement standard and the R coordinate; Table S3: Correlation between each device’s measurement standard and the G coordinate; Table S4: Correlation between each device’s measurement standard and the B coordinate; MS6: Coating material. Whiteness index; Figure S6: Reflectance spectrum of: (A) titanium oxide and (B) barium sulphate; Figure S7: (A) Reflectance spectrum and (B) whiteness index as a function of the number of barium sulphate layers applied to the 3D-printed material; Table S5: Reflectance of barium sulphate in layer 7 of three different devices; Figure S8: Reflectance spectra with and without the application of a protective varnish; MS7: Effect of the illuminant on colour measurements; Figure S9: Normalised spectral distribution of three light sources; Figure S10: Images taken using the W54L511P light source; Figure S11: Images taken using the YJHSMY light source; Figure S12: Images taken using the SMD2835 50S light source; MS8: Electrical stability of the lighting; Figure S13: Comparison of lux levels inside the integrating sphere of the DUNI device over time; Figure S14: Box plot of the R values for different RAL standards measured over 30 min with and without a current stabiliser. MS9: Derivation of the quantitative mathematical model; MS10: Determination of cadaverine; Figure S15: Graphical representations of the (R_0_ – R)/R_0_ values as a function of the cadaverine concentrations in Quantofix^®^ commercial strips; Figure S16: Effect of TMB; Figure S17: Effect of PUO; Figure S18: Graphical representations of the (R_0_ – R)/R_0_ values as a function of the cadaverine concentrations in cellulose-based supports MS11: Other applications using cellulose-based supports; Figure S19: Graphical representations of the (R_0_ – R)/R_0_ values as a function of the Tyramine concentrations in cellulose-based supports; Figure S20: Graphical representations of the (G_0_ – G)/G_0_ values as a function of the Peroxide concentrations in cellulose-based supports; Figure S21. Graphical representations of the (G_0_ – G)/G_0_ values as a function of the cadaverine concentrations in cellulose-based supports.

## Abbreviations

The following abbreviations are used in this manuscript:

MSLA	Masked Stereolithography Technology
HRP	Peroxidase from Horseradish
AB	Biogenic Amines
AO	Amino Oxidase
TMB	3,3,5,5-Tetramethylbenzidine
AR	10-Acetyl-3,7-dihydroxyphenoxazine
PUO	Putrescine Oxidase
TAO	Tyramine Oxidase
PETG	Polyethylene Terephthalate Glycol

## References

[1] Yang F.Q., Ge L. (2023). Colorimetric Sensors: Methods and Applications. Sensors.

[2] Lee Y., Haizan I., Sim S.B., Choi J.H. (2025). Colorimetric Biosensors: Advancements in Nanomaterials and Cutting-Edge Detection Strategies. Biosensors.

[3] Behboudi E., Khadivi-Derakhshan S., Pirouzmand M., Jouyban A., Soleymani J. (2025). Spectrophotometric and Smartphone-Based Colorimetric Methods Utilizing Polyvinylpyrrolidone-Capped Silver Nanoparticles for Determining Doxorubicin in Human Plasma Samples. Sci. Rep..

[4] Elagamy S.H., Adly L., Abdel Hamid M.A. (2023). Smartphone Based Colorimetric Approach for Quantitative Determination of Uric Acid Using Image J. Sci. Rep..

[5] Rajbongshi B., Nickhil C., Deka S.C. (2025). Colorimetric Sensor Technologies for Quality Detection in Grains: A Comprehensive Review. J. Food Meas. Charact..

[6] Barseem A., Obaydo R.H., Elagamy S.H. (2025). Smartphone-Based Colorimetric Determination of Imeglimin Hydrochloride Using Eosin Y: A Simple and Eco-Friendly Analytical Approach. Anal. Sci. Adv..

[7] Sudewi S., Li C.H., Penki V.S.S., Zulfajri M., Meitei N.J., Huang G.G. (2024). Colorimetric and Smartphone-Based Dual-Mode Rapid Detection of Congo Red Using Iron Oxide Quantum Dots. ACS Omega.

[8] Hernández Cruz A., Santacruz Ortega H. (2023). Detección de Metales En Agua a Través de Teléfonos Inteligentes. Epistemus.

[9] Ponhong K., Nilnit T., Lee C.Y., Kusakunniran W., Saetear P., Supharoek S.A. (2025). A Facile Smartphone-Based Digital Image Colorimetric Sensor for the Determination of Tetracyclines in Water Using Natural Phenolic Compounds Induced to Grow Gold Nanoparticles. RSC Adv..

[10] Fyfe C., Yu S., Zhang J., Reid M. (2025). RGB Color Correction and Gamut Limitations in Smartphone-Based Kinetic Analysis of Chemical Reactions. Anal. Bioanal. Chem..

[11] Hijazi A., Al-Masri A., Rawashdeh N. On the Use of Bayer Sensor Color Cameras in Digital Image Correlation. Proceedings of the 11th International Symposium on Signal, Image, Video and Communications, ISIVC 2022—Conference Proceedings.

[12] Cebrián P., Pérez-Sienes L., Sanz-Vicente I., López-Molinero Á., de Marcos S., Galbán J. (2022). Solving Color Reproducibility between Digital Devices: A Robust Approach of Smartphones Color Management for Chemical (Bio)Sensors. Biosensors.

[13] Ulucan O., Ulucan D., Ebner M. (2026). A Traditional Approach for Color Constancy and Color Assimilation Illusions with Its Applications to Low-Light Image Enhancement. Int. J. Comput. Vis..

[14] Xianming L., Ting Z., Yuling X., Yi Z., Peng W. (2024). Field-Deployable Colorimetric Array for On-Site Diagnosis of Urinary Tract Infection and Identification of Causative Pathogens. Anal. Chem..

[15] Shukla S., Sah A.N., Hatiboruah D., Ahirwar S., Nath P., Pradhan A. (2022). Design, Fabrication and Testing of 3D Printed Smartphone-Based Device for Collection of Intrinsic Fluorescence from Human Cervix. Sci. Rep..

[16] Li B., Li L., Guan A., Dong Q., Ruan K., Hu R., Li Z. (2014). A Smartphone Controlled Handheld Microfluidic Liquid Handling System. Lab Chip.

[17] Hossain A., Canning J., Ast S., Rutledge P.J., Yen T.L., Jamalipour A. (2015). Lab-in-a-Phone: Smartphone-Based Portable Fluorometer for PH Measurements of Environmental Water. IEEE Sens. J..

[18] Bui T.H., Thangavel B., Sharipov M., Chen K., Shin J.H. (2023). Smartphone-Based Portable Bio-Chemical Sensors: Exploring Recent Advancements. Chemosensors.

[19] Heidari-Bafroui H., Ribeiro B., Charbaji A., Anagnostopoulos C., Faghri M. (2021). Portable Infrared Lightbox for Improving the Detection Limits of Paper-Based Phosphate Devices. Measurement.

[20] Kim S.D., Koo Y., Yun Y. (2017). A Smartphone-Based Automatic Measurement Method for Colorimetric PH Detection Using a Color Adaptation Algorithm. Sensors.

[21] Vargas-Muñoz M.A., Morales J., Cerdà V., Ferrer L., Palacio E. (2023). Paper Sensor-Based Method Using a Portable 3D-Printed Platform and Smartphone-Assisted Colorimetric Detection for Ammonia and Sulfide Monitoring in Anaerobic Digesters and Wastewater. Microchem. J..

[22] Cooksongold Comercial Lightbox. https://www.cooksongold.com/category_select.jsp?channel=uk&query=video+and+photography+box&filtercategories=catalog01_cooksongold_jewellery2&filterrange=photo_light_box&fh_refpath=b6965d81-fd9a-4b80-8b05-5ec4e38334fb.

[23] Jelken J., Brall T., Gelbing P., Foschum F., Kienle A. (2024). Characterization of the Optical Properties of Photoluminescent Turbid Media Using an Integrating Sphere and Monte Carlo Simulations. Materials.

[24] Vaselnia S.Y., Khajeh Aminian M. (2024). Review on the Application of Density Functional Theory to Predict the Color, Electronic, and Optical Properties of Ceramic Pigments along with Experimental Confirmation. J. Mater. Sci..

[25] Sandilands L.J., Cameron T. (2023). Integrating Sphere Port Error in Diffuse Reflectance Measurements. Appl. Opt..

[26] Gigaherzt Optik GmbH Integrating Sphere for Universal Use in Reflection, Transmission and Absorption Measurements. https://www.gigahertz-optik.com/es-es/producto/upb-150-arta/.

[27] Opsytec Dr. Gröbel GmbH Integrating Spheres—For Measurement and as Homogeneous Light Sources. https://www.opsytec.com/products/integrating-spheres.

[28] Muhammad R., Lee S.H., Htun K.T., Nettey-Oppong E.E., Ali A., Jeong H.W., Seok Y.S., Kim S.W., Choi S.H. (2023). Customized Integrating-Sphere System for Absolute Color Measurement of Silk Cocoon with Corrugated Microstructure. Sensors.

[29] BaySpec Handheld VIS-NIR-SWIR Spectrometer. https://www.bayspec.com/products/handheld-spectrometer-integrating-sphere/?utm_source=chatgpt.com.

[30] Sanz-Vicente I., Rivero I., Marcuello L., Montano M.P., de Marcos S., Galbán J. (2023). Portable Colorimetric Enzymatic Disposable Biosensor for Histamine and Simultaneous Histamine/Tyramine Determination Using a Smartphone. Anal. Bioanal. Chem..

[31] Sanz-Vicente I., López-Molinero Á., de Marcos S., Navarro J., Cebrián P., Arruego C., Visiedo V., Galbán J. (2020). Smartphone-Interrogated Test Supports for the Enzymatic Determination of Putrescine and Cadaverine in Food. Anal. Bioanal. Chem..

[32] Connah D., Westland S., Thomson M.G.A. (2001). Recovering spectral information using digital camera systems. Color. Technol..

[33] Labsphere, Inc. (2017). Integrating Sphere Theory and Applications (Technical Guide). Labsphere. https://www.labsphere.com/wp-content/uploads/2021/09/Integrating-Sphere-Theory-and-Applications.pdf.

[34] Schneider C.A., Rasband W.S., Eliceiri K.W. (2012). NIH Image to ImageJ: 25 years of image analysis. Nat. Methods.

[35] Di Pinto V., Gibertoni G., Rovati L. Investigation of a High-Reflectance Coating for Wide-Spectrum Visual Stimulation. Proceedings of the 2025 Imeko TC2 International Symposium Modern Photonic Metrology.

[36] Homer L., Targowski P., Kowalska M., Iwanicka M., Rosi F., Cartechini L., Buti D., Liang H., Cheung C.S., Liggins F. (2026). Non-invasive characterisation of varnished modern paintings: Comparative insights and conservation implications. J. Cult. Herit..

[37] Kubelka P., Munk F. (1931). An article on optics of paint layers. Z. Tech. Phys..

[38] Thennadil S.N. (2008). Relationship between the Kubelka-Munk scattering and radiative transfer coefficients. Opt. Soc. Am. A.

[39] Hubbe M.A. (2008). Paper’s appearance: A review. BioResources.

