# Supplementary material for: DUNI: A Portable Smartphone-Coupled Integrating Sphere for Controlled Illumination and Reliable Colorimetric Sensing: Analytical Applications
Source: Sensors (Basel). 2026 May 24;26(11):3329. doi: 10.3390/s26113329 (PMC13259253; doi:10.3390/s26113329)
Supplement: Supplementary file 1 [file sensors-26-03329-s001.zip › sensors-4274560-supplementary.pdf]

sensors

Supplementary Material

DUNI: A Portable Smartphone-Coupled Integrating Sphere for Controlled Illumination and Reliable Colorimetric Sensing: Analytical Applications

Pablo Cebrián [1], José Manuel Escuín [1,†], Jesús Salafranca [2], Carmen Jarne [1,3], Ángel López-Molinero [1], Susana de Marcos [1,3], Javier Galbán [1,3] and Isabel Sanz-Vicente [1,3,*]

[1] Nanosensors and Bioanalytical Systems (N&SB), Analytical Chemistry Department, University of Zaragoza, 50009 Zaragoza, Spain; cebrianpab@unizar.es (P.C.); gba@unizar.es (J.M.E.); mjarne@unizar.es (C.J.); anlopez@unizar.es (Á.L.-M.); smarcos@unizar.es (S.d.M.); jgalban@unizar.es (J.G.)
[2] Analytical Chemistry Department, Aragon Institute of Engineering Research (I3A), University of Zaragoza, 50018 Zaragoza, Spain; fjsl@unizar.es
[3] Aragon Institute of Nanomaterials (INMA), University of Zaragoza-Consejo Superior de Investigaciones Cienctificas CSIC, 50018 Zaragoza, Spain
* Correspondence: isasanz@unizar.es
† Current address: School of Engineering and Technology (ESIT), International University of La Rioja (UNIR), Avenida de la Paz, 137, 26006 Logroño, Spain.

MS1: Device design and auxiliary components

A. Device Design

Figure S1 shows a cross-sectional view of the device.

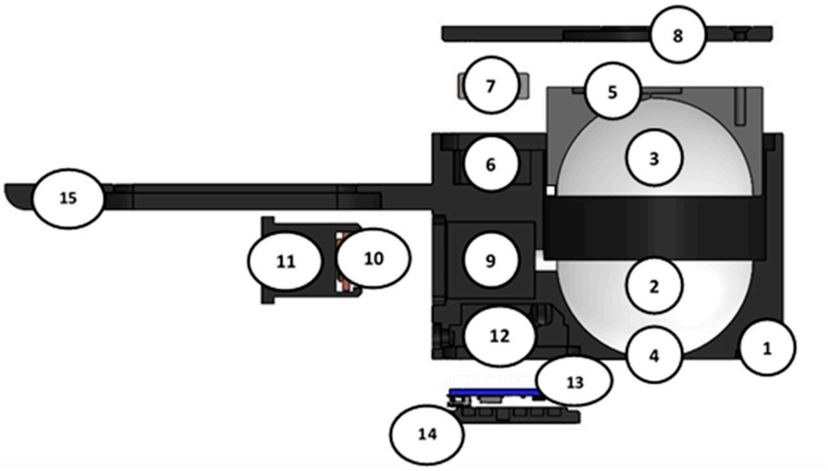

Figure S1. Cross-section of the DUNI device. This figure shows the different parts of the structure designed to house the electronic components, as well as the integrating sphere.

The assembly consists of several components arranged around a main structure (1) that serves as the device's chassis. This structure incorporates the lower hemisphere of the integrating sphere (2) as an integral part, facilitating its assembly with the upper hemisphere (3). At the bottom of the structure there is a hole (4) designed to place the device directly on the sample to be measured, while at the top there is a hole (5) that allows the color samples to be viewed.

 https://doi.org/10.3390/xxxxx

The main structure has several internal compartments specifically designed for the integration of electronic components. In the upper section, there is a compartment (6) for the battery (7). In the central section, there is a compartment (8) for the LED (9), which illuminates the interior of the integrating sphere. Finally, at the bottom there is a compartment (12) for the charging module (13), which acts as a connection point for the rest of the device's electronic components. This module is integrated into the main structure via a bottom cover (14) that ensures its mechanical protection.

The device is completed with several functional covers. A side cover (9) allows the printed circuit board (PCB) placement, which supports the LED, ensuring its proper alignment with the opening that connects to the integrating sphere. The top cover (8) seals the device, providing protection for both the battery and the upper integrating hemisphere.

The device is designed so that measurements can be taken by placing any smartphone on top of it, aligning the smartphone's camera with hole 5. To accommodate any smartphone, the device features a protrusion (15) onto which a universal smartphone adapter can be attached, allowing the smartphone to be secured in place and its camera aligned with hole 5. The universal adapter is attached to part 15 using an M5 screw.

A series of auxiliary components were developed that attach to DUNI to enable analytical measurements both in solution and on commercial test strips (or on cellulose substrates developed in the laboratory).

B. Holder for commercial test strips:

It consists of a rigid plastic strip with the reactive zone attached to one end. The manufactured part consists of two components: a base for holding the strip and a closure cap with a small hole for injecting the reagents onto the strip's paper. The base has two guides for sliding and positioning the cap. Both the component parts and their assembly can be seen in Figure S2A.

Initially, they were constructed using the same polymer resin as DUNI; however, during a blank test with a commercial strip, interaction between the material and the dye was observed, so it was replaced with Polyethylene Terephthalate Glycol filament (PETG). Figure S2B shows the variation of the R coordinate over time in a blank test using a test strip holder made of the polymer resin and PETG.

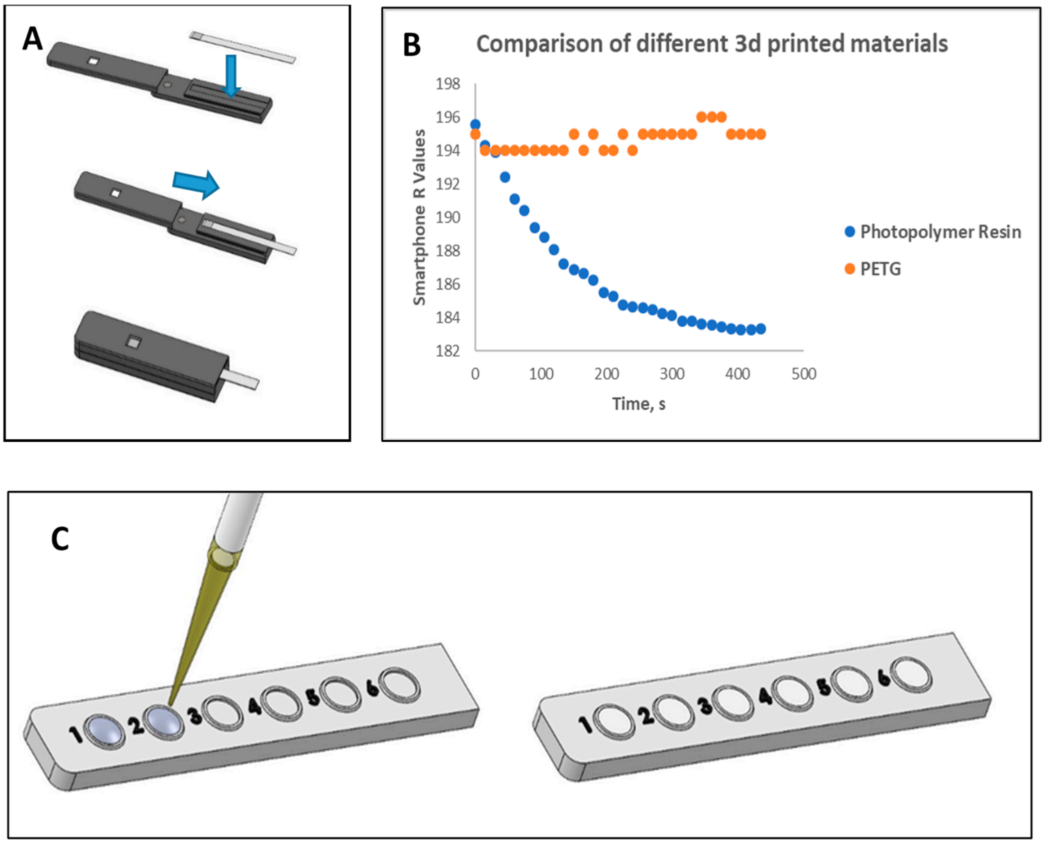

Figure S2. (A) Component parts and assembly of the holder for commercial test strips, (B) Variation of the R coordinate over time in a blank test using a test strip holder made of the polymer resin and PETG and (C) Preparation of cellulose supports.

C. Holder for cellulose supports

It consists of a PETG strip with 6 wells for injecting up to 50 µL of a mixture of cellulose and reagents (Figure S2C). Once injected, the wells are dried in an oven, creating a reaction medium for chemical reactions.

MS2: Need for a position stabilizer

The first prototypes did not have a stabilizer system, as shown in Figure S1, and the smartphone was placed on top of the device. When studying the reproducibility of the measurements, significant variability was observed, as can be seen in Figure S3A, which shows the superimposition of five replicates of an image. To avoid this lack of reproducibility, a stabilizer was designed (Figure S1) that allows any smartphone to be placed and secured, regardless of the camera's position. Figure S3B shows the photographs of five replicates using the stabilizer. The average RGB values and standard deviation are shown in Table S1, where it can be seen that the average value remains constant whilst the standard deviation decreases with the use of the stabiliser.

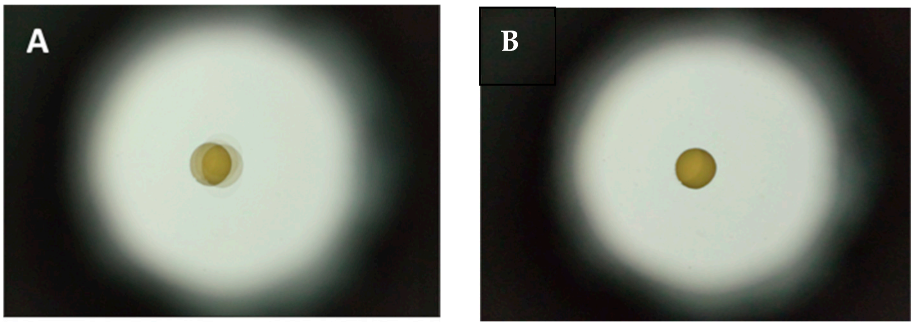

Figure S3. Superimposition of 5 images taken: (A) without a stabilizer and (B) with a smartphone stabilizer. Photographic settings: Auto mode – enabled and focal length – 10 mm.

Table S1. Measurements of five replicas of a RAL standard, with and without a smartphone stabilisation system.

	Without Stabilizer			With Stabilizer		
	R	G	B	R	G	B
Media	155.2	141.0	60.8	155.0	138.1	59.3
s	7.1	7.9	7.9	0.9	1.1	1.1

MS3: Angle effect

Integrating spheres typically use different configurations depending on the type of measurement to be performed [33]. The most common configurations are described below (Figure S4):

- 0/d geometry: In this configuration, the sample is illuminated at 0° (normal incidence) using directional light, and the reflected radiation is collected diffusely by an integrating sphere. The detector receives the hemispherical reflected flux, which includes both diffuse and specular components.
- d/0 geometry: The sample is illuminated diffusely by the inner walls of the integrating sphere, ensuring uniform irradiation from all directions. The detector is positioned at 0° relative to the sample normal. In this geometry, both diffuse and specular components can contribute to the measured signal, although the specular contribution may be partially controlled depending on the optical design (e.g., baffles or traps).
- 8/d geometry: The sample is illuminated directionally at 8° relative to the normal, and the reflected light is collected diffusely by the integrating sphere. This configuration typically includes the specular component (SCI), as specular reflections are captured within the sphere. A specular trap can be introduced to exclude this component (SCE).
- d/8 geometry: The sample is illuminated diffusely by the integrating sphere, and the detector is positioned at 8° relative to the normal. This geometry allows measurement of both diffuse and specular reflectance depending on the configuration. The specular component can be included (SCI) or excluded (SCE) using a specular trap, making this geometry widely used in industrial color measurement standards.

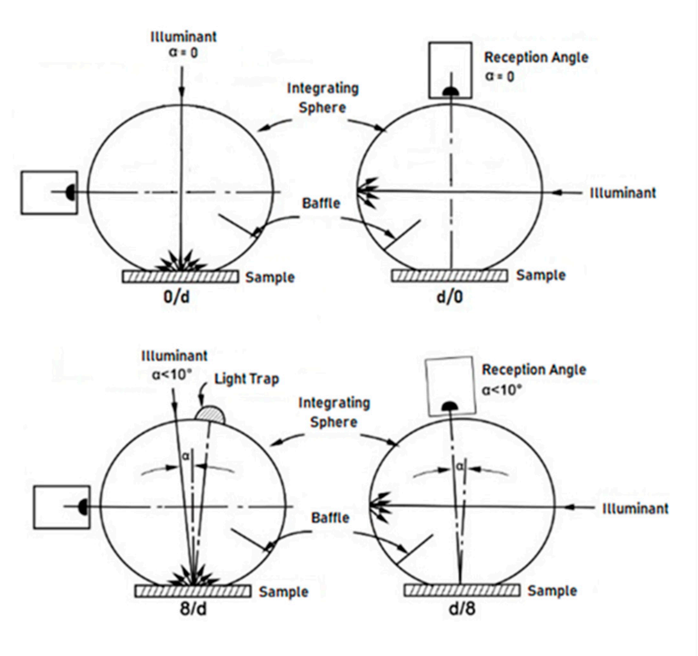

Figure S4. Most common geometries used for integrating spheres. The relative positions of the sample, sensor and light source determine the type of measurements taken and the results obtained.

As mentioned above, the internal geometry of the integrating sphere determines the type of measurements taken by the detector; however, if the sensor is not positioned at an optimal angle relative to the sample, a number of effects may arise that affect the accurate reading of the sample's color, such as:

- Direct view effect: if the sensor is too closely aligned with the sample, direct light may be captured instead of diffuse light, which reduces proper signal integration and can lead to overestimated reflectance.
- Shadow interference: depending on the angle between the sensor and the sample, part of the light flux may be blocked, affecting the uniform distribution of illumination within the sphere.
- Aperture loss effect: an incorrect angle or an aperture that is too large on the sensor can cause some of the light to be lost, altering the sphere's efficiency and reducing the accuracy of the measurement.

It is for all these reasons that the aim with an integrating sphere is to optimize the relative position between the sensor and the sample according to the type of color sample to be measured. For the development of the device, the configurations studied were d/0 and d/8.

MS4: ImageJ v 2.3.0/1.53t software to study color homogeneity

To investigate the effect of the angle between the aperture through which the detector is positioned and the aperture placed over the sample, an image analysis was carried out using the ImageJ software [34].

This programme allows you to analyse a specific area of the image and generate a representation of the pixel intensity – or amount of light – within that area using a tool called Surface plot. To aid interpretation, the programme also allows a colour palette to be used to distinguish these areas:

- Red or yellow colors: areas of high intensity or bright light
- Blue or purple colors: areas of low intensity or low light

Figure S5 shows a photograph of the same RAL taken with the d/0 (A) and d/8 (B) devices and the result of applying the aforementioned processing to both images (C and D)

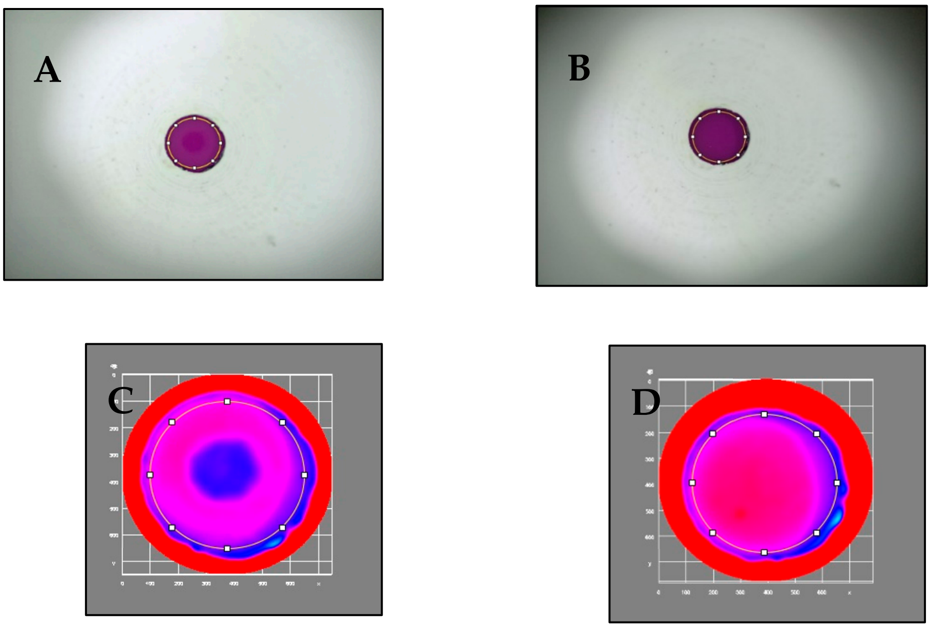

Figure S5. Photograph of a RAL standard taken with the the d/0 (A) and d/8 (B) device, and light distribution across the surface the d/0 (C) d/8 (D).

The central zone shows the greatest differences between the two devices, as in the device with d/0 geometry a blue zone can be observed in the center of the area selected (Figure S5C), whilst in the d/8 geometry device the zone is more homogeneous (Figure S5D). This is because the upper aperture of the sphere (where the smartphone camera is positioned) is reflected onto the sample, distorting the RGB reading values of the color sample. The average intensity in the central area of the color samples was 47.52 for d/0 and 52.30 for d/8, with a standard deviation of 5.64 and 0.65 respectively, demonstrating that the device's angle affects the measurements.

MS5: Integrating sphere size

Twenty-four RAL colour standards were measured for each sphere, recording the three coordinates. For each coordinate and each sphere, the relationship between the measured coordinates and the actual values was plotted. The y-intercepts and slopes of the resulting plots are shown in Table S2, S3 and S4, along with the correlation coefficients.

Table S2. Correlation between each device's measurement-standard and the R coordinate

**Φ (mm)**	**Slope**	**y-intercepts**	**R^2**
**33**	0.6630	44.0566	0.9873
**36**	0.7092	46.1319	0.9903
**39**	0.7189	49.6038	0.9897
42	0.7293	51.3442	0.9911

Φ: internal diameter of sphere

Table S3. Correlation between each device's measurement-standard and the G coordinate

**Φ (mm)**	**Slope**	**y-intercepts**	**R^2**
**33**	0.7777	11.9060	0.9873
**36**	0.7875	15.8048	0.9900
**39**	0.7895	17.7833	0.9875
42	0.8085	22.3412	0.9910

Φ: internal diameter of sphere

Table S4. Correlation between each device’s measurement-standard and the B coordinate

**Φ (mm)**	**Slope**	**y-intercepts**	**R^2**
**33**	0.5829	74.2038	0.9737
**36**	0.6027	75.3970	0.9867
**39**	0.6150	78.6053	0.9861
42	0.6573	80.0708	0.9896

Φ: internal diameter of sphere

MS6: Coating material. Whiteness index

Based on their optical properties and the relative ease with which they can be applied as a coating on the inside of the sphere [35], two types of coating were selected: $BaSO_4$ and TiO_2.

To evaluate these coatings, aspects such as spectral response, reflectance efficiency and the accuracy of colour pattern measurements will be analysed. Other aspects will also be evaluated, such as the minimum number of coating layers required for optimal light distribution within the sphere, preparation costs and the use of coating protection materials, such as varnishes.

To evaluate the spectral response and reflectance of both materials, several layers of each were applied to a smooth surface and measured using a Konica Minolta CM-2600d spectrophotometer, which measures the reflectance spectrum of a surface and, in the case of white materials, provides an index of the total amount of light they reflect (CIE Whiteness Index). This index quantifies how ‘white’ a material appears under standardised lighting and observation conditions compared to a reference White. The reflectance spectra of both materials are shown below (Figures S6A and S6B):

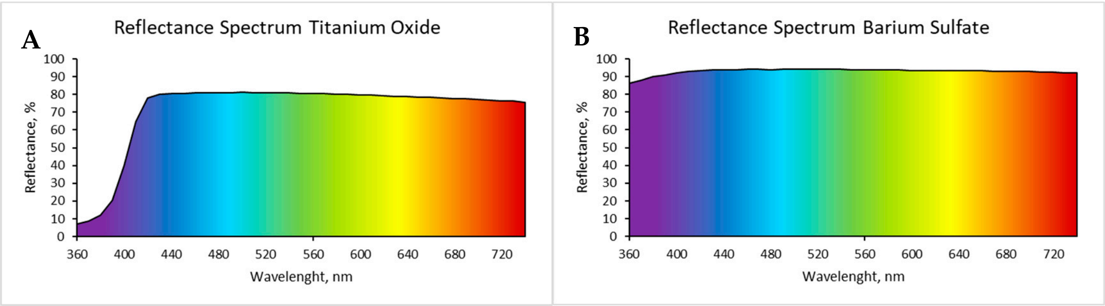

Figure S6. Reflectance spectrum of: (A) titanium oxide and (B) barium sulphate.

As can be seen from the graphs, titanium oxide exhibits an absorption band between 360 and 400 nm, whereas barium sulphate has a more uniform spectrum. If we evaluate the total light reflectance of both materials, titanium oxide would around 89 % reflectance, compared to 97% for barium sulphate. All this data leads to the conclusion that barium sulphate is a much more suitable material for coating the integrating sphere, as it reflects a much greater amount of light and has more uniformly across the entire visible spectrum.

A. Number of coating layers

The next step was to assess the effect of the number of layers of reflective material required to cover the interior of the integrating sphere evenly. To this end, a series of rectangular pieces were first 3D printed, onto which different numbers of layers of reflective material were applied using an airbrush (a total of 8 layers), and the reflectance spectrum of these pieces was measured in triplicate to ensure that the airbrush coating could be applied in a reproducible manner. The results obtained for the reflectance spectra and whiteness index as a function of layer thickness are shown in Figures S7: as the number of layers increases, both the reflectance and the whiteness index increase until reaching a maximum at 7 layers.

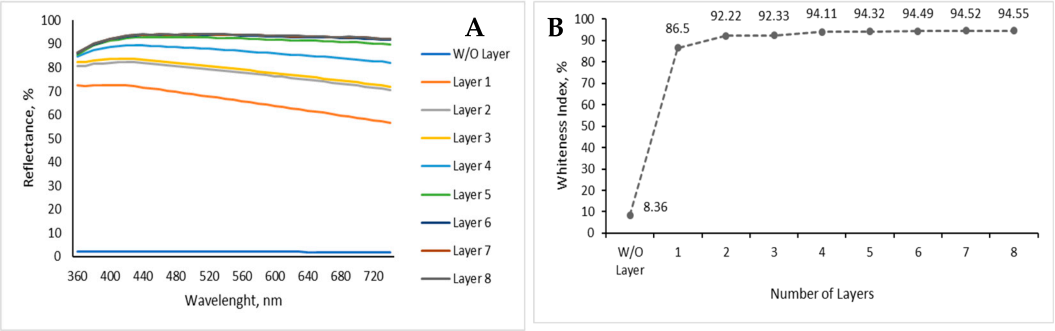

Figure S7: (A) Reflectance spectrum and (B) whiteness index as a function of the number of barium sulphate layers applied to the 3D-printed material. Each data point in both graphs represents the average of three measurements.

To verify the reproducibility of both 3D printing of the devices and the internal coating of the integrating sphere, three DUNI devices were printed and subjected to the same barium sulphate application procedure; the reflectance spectra were then measured and a one-way ANOVA test was performed, using layer number 7 of the three devices as a reference (table S5). After applying the test, the results showed that there were no statistically differences between the three data sets, as the p-value (0.9994) is greater than 0.05; therefore, the null hypothesis of equal means across the groups is not rejected.

Finally, the coating thickness was measured using a Mitutoyo micrometer, yielding a value of 0.05 mm. This data will enable the preparation and coating costs to be calculated for inclusion in the total manufacturing costs of the DUNI device.

Tabla S5. Reflectance of barium sulphate in layer 7 of three different devices that have been spray-coated

nm	Average	s	nm	Average	s
380	94.22	0.09	570	97.61	0.09
390	95.07	0.09	580	97.70	0.10
400	96.37	0.09	590	97.36	0.11
410	96.74	0.09	600	97.50	0.09
420	97.60	0.09	610	97.31	0.10
430	97.72	0.09	620	97.30	0.10
440	98.07	0.09	630	97.02	0.10
450	97.80	0.09	640	97.15	0.10
460	98.14	0.09	650	96.96	0.10
470	97.92	0.09	660	97.09	0.09
480	98.06	0.09	670	96.79	0.11
490	98.05	0.09	680	96.90	0.09
500	98.16	0.09	690	96.68	0.11
510	98.02	0.09	700	96.77	0.09
520	98.19	0.09	710	96.32	0.11
530	97.87	0.09	720	96.36	0.09
540	98.03	0.09	730	96.09	0.09
550	97.80	0.09	740	96.08	0.10
560	97.87	0.09			

B. Use of varnish as a coating protector

Varnishes are transparent coating materials, consisting of mixtures of resins, solvents and various additives, commonly used to protect surfaces (paint, wood, metal, etc.), thereby improving their mechanical and chemical properties. These materials form a thin film between the surface and the air, protecting it from moisture, dust and exposure to sunlight [36].

These materials appear ideal for protecting the internal coating of the DUNI device, as they improve its mechanical properties and long-term durability, preventing the coating from peeling off due to impacts or becoming soiled, which could result in a loss of reflected light within the device.

The following study focuses on the use of a varnish to protect the internal coatings of the sphere and whether they have any effect on the optical properties of the materials used. To this end, the reflectance spectrum of a device coated with barium sulphate was measured before and after applying varnish. The results are shown in Figure S8:

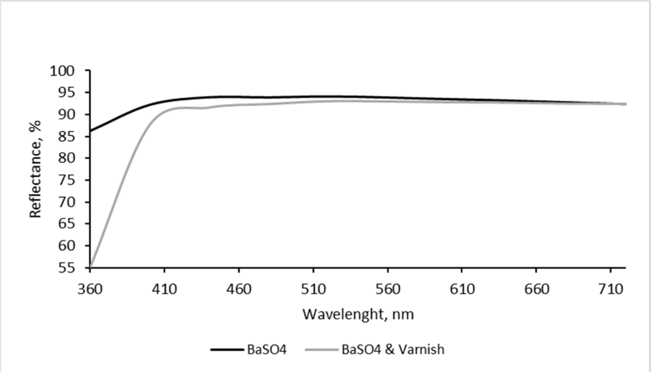

Figure S8: Reflectance spectra with and without the application of a protective varnish

As can be seen, the application of the protective varnish affects the total amount of light that can be reflected by the material only in the near-ultraviolet region; consequently, it was decided to use varnish in the construction of the final device.

MS7: Effect of the illuminant on colour measurements

The influence of the illuminant on colour measurements was investigated. Three LED illuminants were selected: W54L511P, YJHSMY and SMD2835 50S, whose spectral power distributions are shown in Figures S9 A, B and C respectively. The main differences between them lie in the relative intensity of the blue region and the continuity of the spectrum.

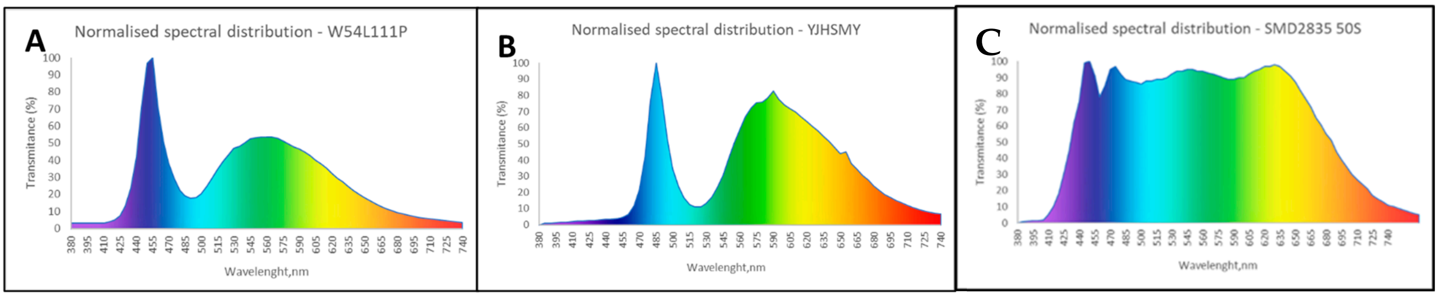

Figure S9: Normalised spectral distribution of three light sources: (A) W54L111P, (B) YJHSMY and (C) SMD2835 50S.

Fifty-five standardised RAL colour samples were measured using the three illuminants installed in the DUNI device. The photographs are shown in Figures S10, S11 and S12 (Photographic measurement conditions: Auto mode – Enabled and focal length – 10 mm), and the results are discussed in the main article

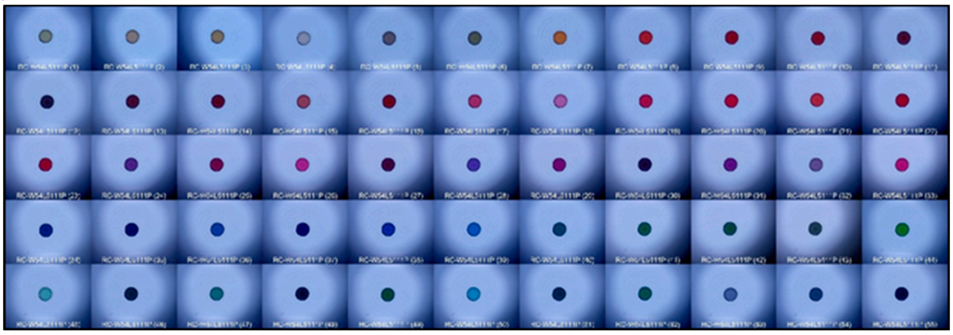

Figure S10: Images taken using the W54L511P light source.

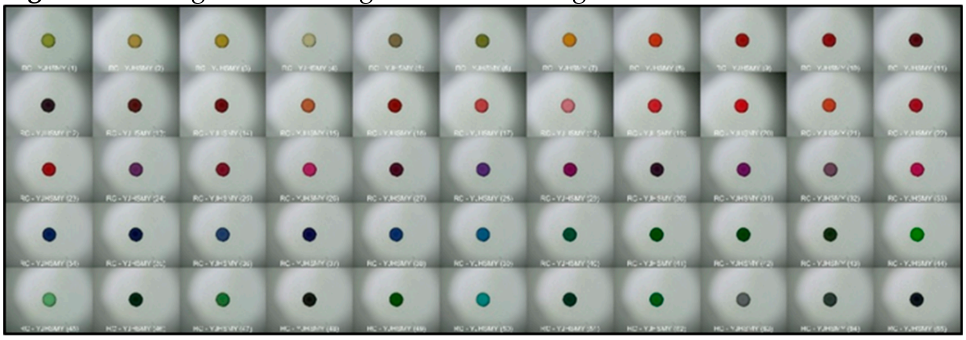

Figure S11: Images taken using the YJHSMY light source.

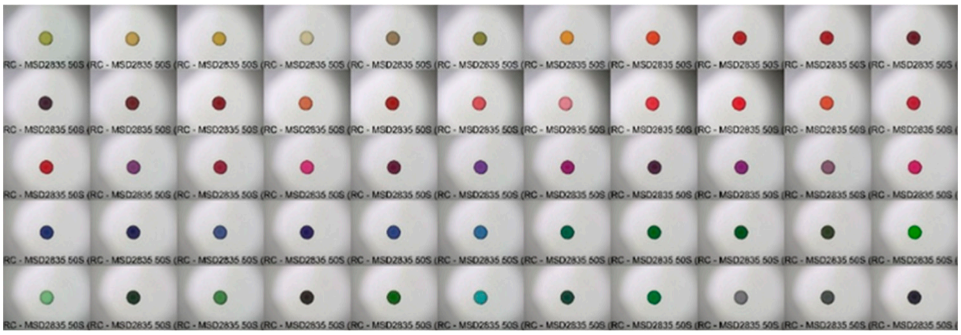

Figure S12: Images taken using the SMD2835 50S light source.

MS8: Electrical stability of the lighting

To study the electrical stability of the lighting, a lux meter was placed in the lower opening of the device. With the battery fully charged (1000 mAh), the SMD2835 50S was switched on and measurements were recorded every hour (Figure S13A). It can be seen that the progressive discharge of the battery causes a continuous decrease in the supplied current, which is reflected in a decay of the luminous intensity with a potential-type behaviour. This phenomenon not only affects the lux value, but may also affect the variability of the colorimetric measurements, introducing a source of error.

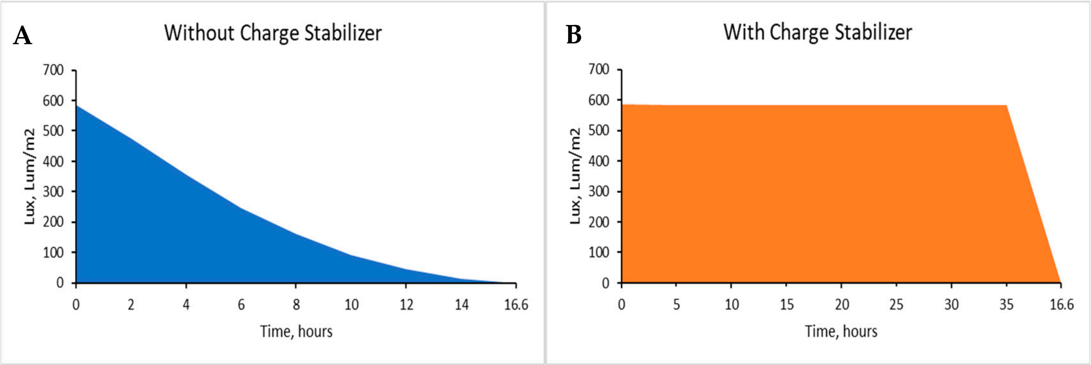

Figure S13: Comparison of lux levels inside the integrating sphere of the DUNI device over time: (A) without a charge stabiliser (B) with a charge stabiliser.

The same experiment was carried out, but applying modifications to the PCB housing the LED, incorporating a charge stabiliser so that the current supplied to the LED remains practically constant over time. The results are shown in Figure S13B, which demonstrates that the lux level remains stable until, as the battery runs out, there is an abrupt drop, ensuring that the illumination within the sphere is stable and uniform.

To study the effect on the measurements, five blue RAL colour patches of different shades were selected, and their RGB values were measured throughout the battery's service life, using the LED both with and without a charge stabiliser. The images were taken with the smartphone in manual mode to prevent it from compensating for excess or insufficient light when operating in automatic mode. (Figures S14).

As can be seen, the use of an LED with a current stabiliser provides much more stable and controlled measurements, ensuring that they are reliable and reproducible over time.

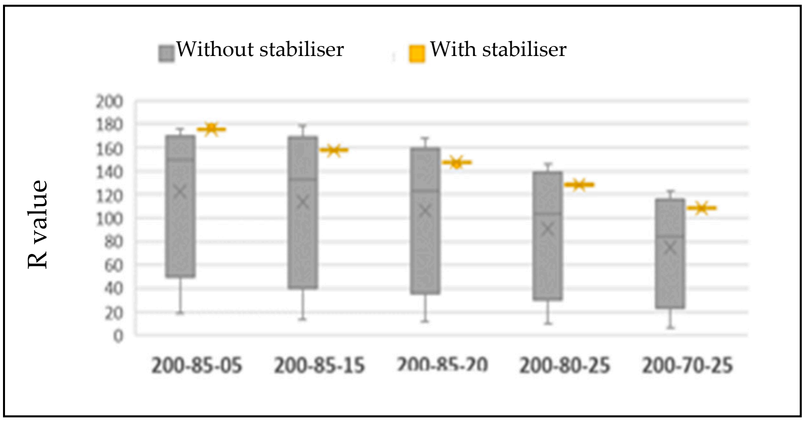

Figure S14: Box plot of the R values for different RAL standards measured over 30 minutes with and without a current stabiliser. The photographs were taken in: Auto Mode – Disabled, ISO – 100, Shutter speed – 1/250 s and focal length – 10 mm.

MS9: Derivation of the quantitative mathematical model.

Starting from the absorption and scattering coefficients:

$$K_{KM,\lambda} = K_\lambda = 2.3 \sum_i \varepsilon_{\lambda,i} C_i \qquad (S1)$$

$$S_{KM,\lambda} = \frac{S_\lambda}{2} \qquad (S2)$$

where Ci and $\varepsilon_{\lambda,i}$ are the concentration and molar absorptivity of each absorbent species in the medium. Considering the most general expression for Rλ derived from the Kubelka-Munk equation:

$$R_\lambda = \frac{Sinh\,(b_\lambda S_{KM,\lambda}\ell)}{a_\lambda Sinh\,(b_\lambda S_{KM,\lambda}\ell) + b_\lambda Cosh\,(b_\lambda S_{KM,\lambda}\ell)} \qquad (S3)$$

with

$$a_\lambda = 1 + \frac{K_{KM,\lambda}}{S_{KM,\lambda}} \qquad b_\lambda = \sqrt{a_\lambda^2 - 1} \qquad (S4)$$

As has been indicated, the experimental results show that Rλ varies with C according to a second-degree polynomial equation, so the application of Taylor series gives:

$$R = \left(\frac{S_\lambda \ell}{2 + S_\lambda \ell}\right) + 9\frac{\ell}{(2 + S_\lambda \ell)^2}\varepsilon_\lambda C + 84\frac{\ell^2}{S(1 + S_\lambda \ell)^3}\varepsilon_\lambda^2 C^2 \qquad (S5)$$

Substituying in equation (8) of main paper:

$$E_{(R,G,B)} = B\frac{f_d}{f_s}\frac{1}{\left(\varphi - R_{(R,G,B)}\right)} \quad (S6)$$

and applying the Taylor expansion:

$$\frac{1}{1 - x} \approx 1 + x + x^2 \; (S7)$$

One obtains:

$$E_{(R,G,B)} = E_{0,(R,G,B)}\,(1 + \alpha C + \beta C^2) \;\; (S8)$$

Where

$$E_{0,(R,G,B)} = B\frac{f_d}{f_s}\frac{1}{\left(\varphi - \frac{S\ell}{S\ell + 2}\right)} \quad (S9)$$

$$\alpha = -\frac{f_s}{\varphi - \frac{S\ell}{S\ell + 2}}\left(\frac{4.6\,\varepsilon}{S}\right)\frac{1+\frac{S\ell}{3}}{\left(1+\frac{2}{S\ell}\right)^2} \quad (S10)$$

$$\beta = \left(\frac{4.6\,\varepsilon}{S}\right)^2\left[\left(\frac{f_s}{\varphi - \frac{S\ell}{S\ell+2}}\right)^2\left(\frac{\left(1+\frac{S\ell}{3}\right)^2}{\left(1+\frac{2}{S\ell}\right)^4}\right) + \left(\frac{f_s}{\varphi - \frac{S\ell}{S\ell+2}}\right)\left(\frac{\left(1+\frac{S\ell}{3}\right)^2 - \frac{S\ell}{6}\left(1+\frac{2}{S\ell}\right)}{\left(1+\frac{2}{S\ell}\right)^3}\right)\right] \quad (S11)$$

Under the thin-medium condition:

$$S\ell \ll 1 \quad (S12)$$

One obtains the approximations

$$\mathrm{E}_{0,(\mathrm{R,G,B})} = \mathrm{B}\frac{f_d}{f_s}\frac{1}{\left(\varphi - \frac{S\ell}{2}\right)} \quad (S13)$$

$$\alpha = -\frac{f_s S\ell^2}{\varphi - \frac{S\ell}{2}}\left(\frac{4.6\,\varepsilon}{4}\right) \quad (S14)$$

$$\beta = \left(\frac{4.6\,\varepsilon}{12}\right)^2\frac{f_s S\ell^3}{\varphi - \frac{S\ell}{2}} \quad (S15)$$

These expressions lead to Equation (15) in the main manuscript.

MS10: Determination of cadaverine

To determine cadaverine using commercial peroxide test strips, 25 microlitres of the 25 µM PUO enzyme are first applied to the strip, allowed to dry, and then 10 microlitres of the analyte are added. The cadaverine calibration is shown in Figure S15:

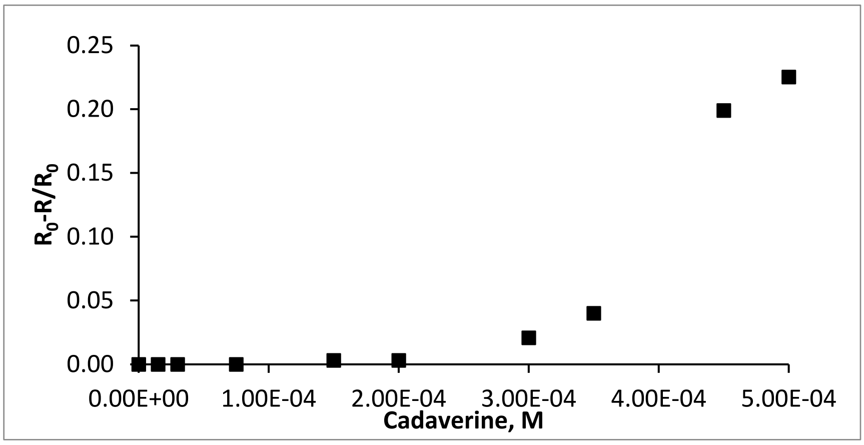

Figure S15: Graphical representations of the $(R_0 - R)/R_0$ values as a function of the Cadaverine concentrations in Quantofix® comercial strips. Conditions: 0.1 M phosphate buffer, pH = 7; 25 µM PUO.

As can be seen from the graph, an increase in the R0-R/R0 values does not become apparent until a cadaverine concentration of $3\cdot10^{-}$[illegible]M, and the signals are very weak compared to those of putrescine. This could be because the cadaverine reaction requires different reaction conditions to those of putrescine; therefore, the pH, HRP concentration and

PUO concentration were investigated. Under none of the conditions studied was a signal obtained for concentrations below $3\cdot10^{-}$◎M.

These results suggested that there might be a reagent in the QF strips that was inhibiting the cadaverine reaction. To verify this, the assay was performed on cellulose-based substrates prepared in the laboratory, as this allows control over the immobilised reagents. For this purpose, the auxiliary support shown in Figures 2F and S2C was used. 75 microlitres of 5% (w/v) cellulose containing TMB were deposited into the mould holes.

These were left to dry, and a mixture of the enzymes PUO and HRP was injected, followed by the analyte. The concentrations of the different reagents were investigated. Figure S16 shows the results of the TMB assay for two concentrations of cadaverine.

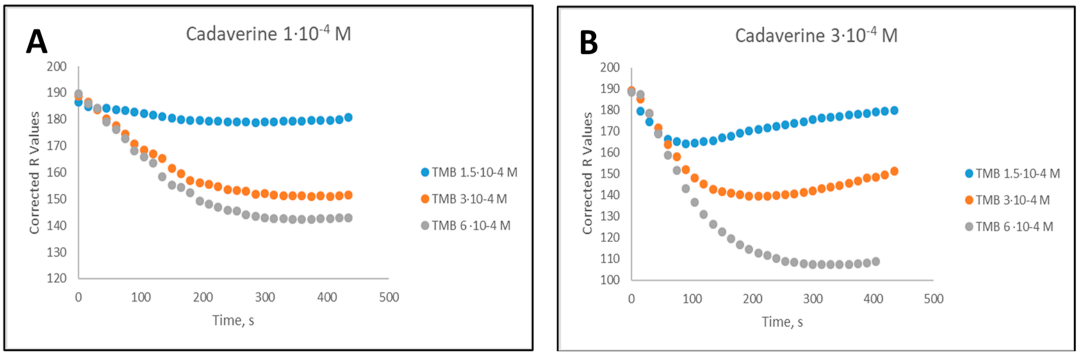

Figure S16: Effect of TMB for: (A) [Cadaverine] = $1\cdot10^{-}$◎M and (B) [Cadaverine] = $3\cdot10^{-}$◎ M. Conditions: 0.1 M phosphate buffer, pH = 7; 10 U/mL HRP; 25 µM PUO.

As can be seen, a signal was detected for cadaverine concentrations of $1\cdot10^{-}$◎M; it is therefore suspected that a reagent in the commercial test strips (the composition of which is unknown) prevented the determination from being carried out.

Furthermore, the amount of immobilised TMB proved to be crucial. The signal variation increases as the TMB concentration rises, and the signal (the colour) remains stable for a longer period. The product of the enzymatic reaction of cadaverine is a strong reductor, so there must be an excess of TMB, as previously demonstrated in a previous article [30]. Figure S17 shows the results of the PUO study for 10, 15 and 25 µM.

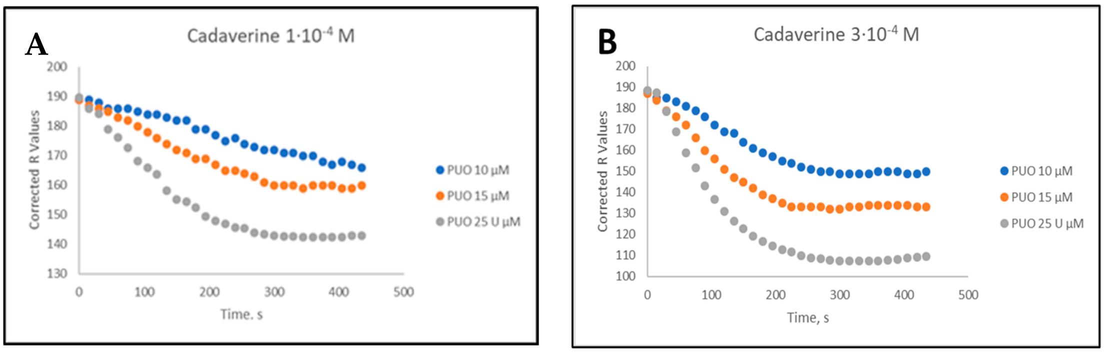

Figure S17: Effect of PUO for: (A) [Cadaverine] = $1\cdot10^{-}$◎M and (B) [Cadaverine] = $3\cdot10^{-}$◎M. Conditions: 0.1 M phosphate buffer, pH = 7; 10 U/mL HRP; TMB $6\cdot10^{-}$◎M

It can be seen from both graphs that the amount of immobilised PUO affects both the signal value obtained and the kinetics of the signal. The optimal amount to immobilise was considered to be 25 µM, as this generated the strongest signal and the duration for which it remained stable was enough to ensure there was no error in the measurement (stable for 2 minutes). As regards the HRP concentration, no effect was observed within the range of 10–24 U/mL. Under optimal conditions, a calibration was performed, yielding the graph shown in Figure S17

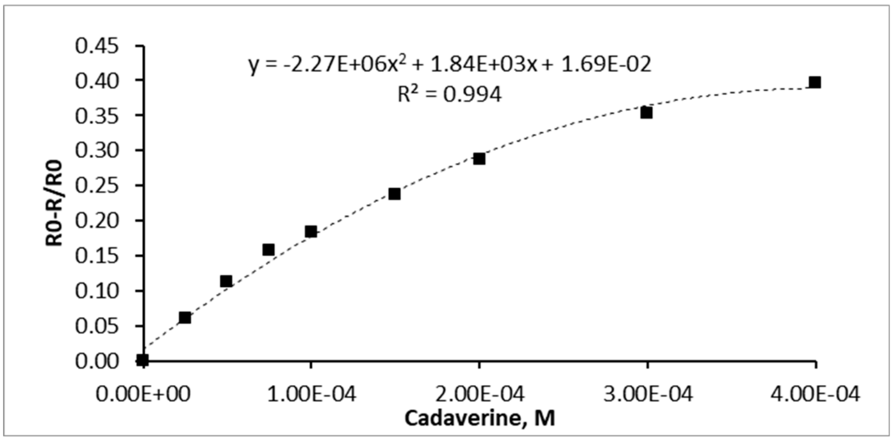

Figure S18: Graphical representations of the (R_0 – R)/R_0 values as a function of the Cadaverine concentrations in cellulose-based supports. Conditions: 0.1 M phosphate buffer, pH = 7; 10 U/mL HRP; 25 µM PUO, TMB $6\cdot10^{-◎}$M.

MS11: Other applications using cellulose-based supports.

A. Inmobilization of Tyramine Oxidase

Other enzymes, such as tyramine oxidase (TAO), can be immobilised, enabling the determination of tyramine. To do this, the cellulose supports are prepared by depositing 75 microlitres of a mixture of 5% cellulose and $6\cdot10^{-◎}$M TMB in 0.1 M phosphate buffer (pH 6) onto the wells of the mould. Once dry, 10 µL of a mixture (16 U/mL TAO and 10 U/mL HRP) is injected, followed by 10 µL of tyramine at different concentrations ranging from $2.5\cdot10^{-5}$ to $3\cdot10^{-◎}$M. The resulting calibration curve is shown in Figure S18:

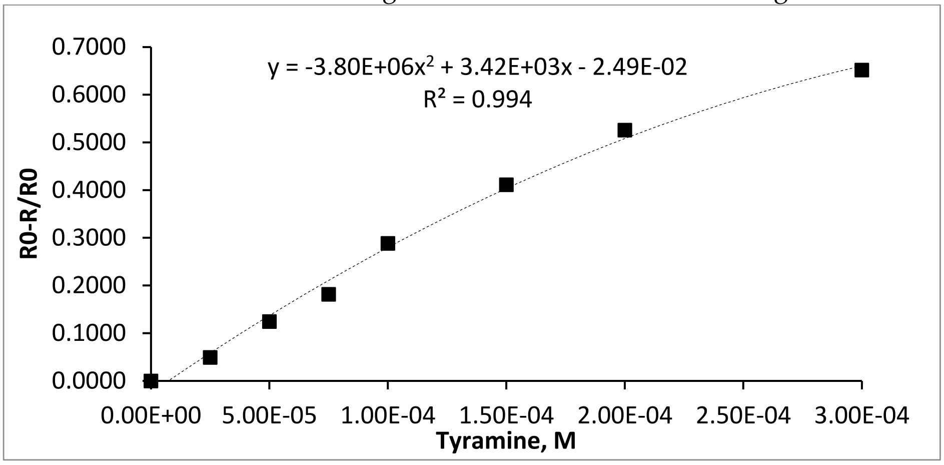

Figure S19. Graphical representations of the R_0 – R)/R_0 values as a function of the Tyramine concentrations in cellulose-based supports. Conditions: 0.1 M phosphate buffer, pH = 6; 10 U/mL HRP; 16 U/mL TAO, TMB $6\cdot10^{-◎}$M

B. Inmobilisation of other dyes

Other dyes can be immobilised, such as Amplex Red, whose oxidation product (resorufin) is pink; consequently, in this case, the G coordinate proved to be more sensitive. Hydrogen peroxide and cadaverine were detected.

For hydrogen peroxide, the cellulose-based supports are prepared by depositing 75 microlitres of the mixture of 5% cellulose and $3\cdot10^{-◎}$M AR in 0.1 M phosphate buffer, pH = 6, onto the holes in the mould. Once dry, 10 µL of 10 U/mL HRP was injected, followed by 10 µL of the analyte. The calibration shown in Figure S19 was obtained.

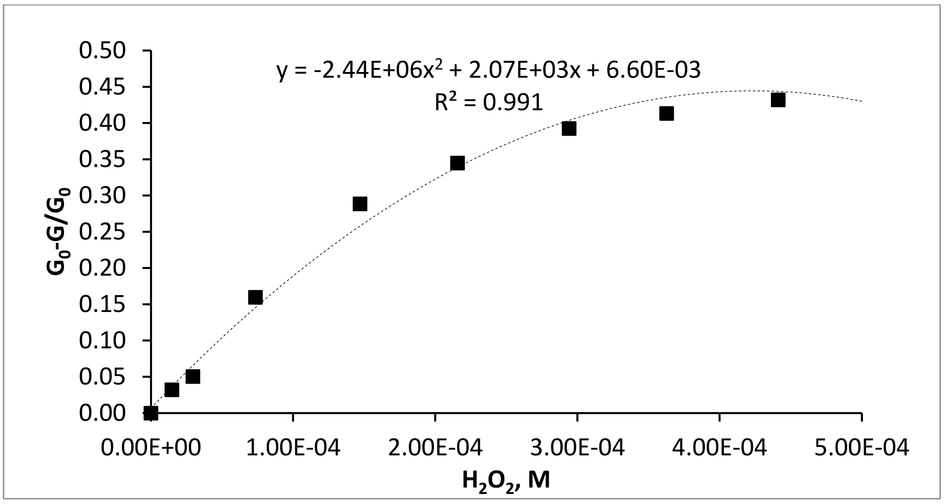

Figure S20: Graphical representations of the ($G_0 - G$)/G_0 values as a function of the Peroxide concentrations in cellulose-based supports. Conditions: 0.1 M phosphate buffer, pH = 6; 10 U/mL HRP; AR 3·10^{-}[illegible]M.

To determine cadaverine, cellulose-based supports are prepared by depositing 75 microlitres of a mixture of 5% cellulose and 3·10^{-}[illegible]M AR in 0.1 M phosphate buffer (pH 7) onto the wells of the mould. Once dry, 10 µL of the mixture of HRP 10 U/mL and PUO 25 µM is injected, followed by 10 µL of the analyte. The calibration curve shown in Figure S20 was obtained.

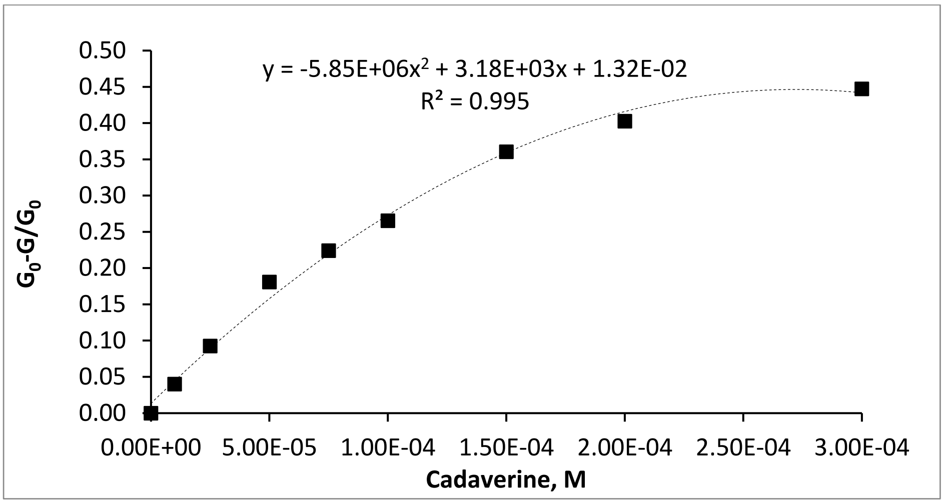

Figure S21: Graphical representations of the ($G_0 - G$)/G_0 values as a function of the Cadaverine concentrations in cellulose-based supports. Conditions: 0.1 M phosphate buffer, pH = 7; 10 U/mL HRP; AR 3·10^{-}[illegible]M, PUO 25 µM

Disclaimer/Publisher's Note: The statements, opinions and data contained in all publications are solely those of the individual author(s) and contributor(s) and not of MDPI and/or the editor(s). MDPI and/or the editor(s) disclaim responsibility for any injury to people or property resulting from any ideas, methods, instructions or products referred to in the content.
